# The SnackerTracker: A novel home-cage monitoring device for measuring food-intake and food-seeking behaviour in mice

**DOI:** 10.12688/wellcomeopenres.23850.1

**Published:** 2025-04-04

**Authors:** Marissa Mueller, Selma Tir, Carina Pothecary, Elise Meijer, Laurence Brown, Keiran Foster, Vladyslav Vyazovskiy, Stuart Peirson, Zoltán Molnár

**Affiliations:** 1 Physiology Anatomy and Genetics, University of Oxford Department of Physiology Anatomy and Genetics, Oxford, Oxfordshire, OX1 3PT, UK; 2 Sleep and Circadian Neuroscience Institute, University of Oxford Sleep and Circadian Neuroscience Institute, Oxford, Oxfordshire, OX1 3QU, UK; 3 Nuffield Department of Clinical Neurosciences, University of Oxford Nuffield Department of Clinical Neurosciences, Oxford, Oxfordshire, OX3 9DU, UK; 4 Kavli Institute for Nanoscience Discovery, University of Oxford Kavli Institute for Nanoscience Discovery, Oxford, Oxfordshire, OX1 3QU, UK; 5 Department of Physics, University of Oxford Department of Physics, Oxford, Oxfordshire, OX1 3PU, UK

**Keywords:** Home-cage monitoring, food measurement, animal behaviour, activity tracking, animal welfare, cryptochrome, circadian, biological rhythms

## Abstract

**Background:**

Accurately measuring activity and feeding is important in laboratory animal research, whether for welfare-monitoring or experimental recording. Quantification commonly involves manual pellet-weighing; however, this can physically disturb animals and cannot continuously assess both the amount and pattern of feeding over time. Improved means of food-intake measurement have been developed but can be costly and incompatible with many cage configurations.

**Methods:**

We developed the *SnackerTracker—*a novel home-cage monitoring system which continuously records food-intake, food-seeking activity, and ambient light conditions in laboratory mice. After benchtop validations, we tested this device by recording from C57BL/6J control mice under 12:12h light:dark (LD) and constant darkness (DD) to measure circadian rhythms in feeding behaviour. We then recorded from mice having disturbed circadian rhythms (cryptochrome 1 and 2 double-knockouts, *Cry1 ^-/-^,Cry2 ^-/-^*), where irregular activity and feeding patterns were expected. Animals were individually housed with *SnackerTrackers* in Digital Ventilated Cages ^®^ (DVC, Tecniplast) to measure home cage activity. After habituation, 48-hour *SnackerTracker* and DVC recordings were collected and compared.

**Results:**

The *SnackerTracker* accurately measured food-masses throughout benchtop and *in vivo* validation tests. Time-course *SnackerTracker* feeding traces correlated well with DVC activity recordings, indicating that feeding reflects general cage locomotion in control and cryptochrome-deficient animals. In LD, *SnackerTracker* data showed expected feeding/fasting cycles in control and cryptochrome-deficient animals yet reduced dark-phase feeding in cryptochrome-deficient mice. In DD, increased feeding during the subjective nighttime was maintained in control animals but abolished in cryptochrome-deficient mice. Surprisingly, cryptochrome-deficient animals exhibited ultradian feeding rhythms.

**Conclusions:**

We validate the performance and value of monitoring home cage feeding using the *SnackerTracker*. Here we show that cryptochrome-deficient animals have decreased food-intake in LD, diurnal arrhythmicity in DD, and ultradian rhythms in feeding behaviour. The *SnackerTracker* provides a cost-effective, open-source, and user-friendly method of animal food intake and activity measurement.

## Plain Language Summary

Accurately measuring activity and feeding is important in laboratory animal research as an experimental outcome or for welfare monitoring. Improved methods have been developed yet are often costly and incompatible with existing cage configurations. To address this issue, Mueller *et al.* created the *SnackerTracker*—a novel open-source home-cage monitoring system which measures the food intake and feeding behaviour of laboratory mice. After conducting extensive validations for accuracy, they tested the *SnackerTracker* with singly-housed wild-type control mice and cryptochrome 1 and 2 double-knockout mice ( *Cry1 ^-/-^,Cry2 ^-/-^*) having disrupted circadian rhythms under both 12:12h light-dark cycles and constant darkness. Irregular activity and feeding patterns were expected in cryptochrome-deficient mice in the absence of regular environmental cues (i.e., constant darkness). Under 12:12h light-dark cycles, as expected, *SnackerTracker* data showed normal nocturnal feeding/fasting cycles in both control and cryptochrome-deficient animals which were abolished in cryptochrome-deficient mice in constant darkness. However, cryptochrome-deficient animals exhibited shorter <24h ultradian rhythms that may be driven by appetite. We show that the *SnackerTracker* can reveal biologically relevant differences in feeding behaviour, providing a low-cost and easy-to-use method of home-cage monitoring in laboratory mice. We provide open-source resources for future applications, emphasising the need for improved hardware/software platforms and open-science.

## Introduction

Home-cage monitoring is increasingly used for both scientific and welfare assessment in animal research ^1, 2^. Food intake measurements and activity-tracking commonly serve as important metrics across basic science and preclinical investigations. Researchers can modify or standardise the type, amount, and timing of food administration to observe effects across animal genotypes, ages, and experimental conditions. Food consumption or changes in feeding behaviour are relevant for metabolic status, psychological state (e.g., stress or pain ^3, 4^), and side effects of pharmacological interventions ^5^. For example, in studies involving transgenic mouse models with known or suspected metabolic phenotypes, knowing the timing and amount of feeding provides insight into whether increased or decreased body weight can be attributed to altered food intake or biological response to the same food intake. This is additionally relevant for welfare monitoring. Prey species like mice may innately mask sickness behaviour, as showing signs of weakness increases susceptibility to predation in nature. Increased levels of inflammatory cytokines during sickness may induce elevated body temperatures, decreased appetite and sleep, and significant metabolic changes leading to weight loss ^6^. Therefore, a cost-effective and customisable system which continuously monitors the amount and timing of feeding may be a useful indication of animal health and well-being and would contribute to ongoing progress in the field of animal home-cage monitoring ^2^.

Manually measuring food-intake typically involves placing pellets of a known mass into cages, later retrieving these pellets, then calculating the mass difference. However, pellets are prone to crumbling which may erroneously inflate measurements. Researchers physically disrupt animal cages by adding and removing food, which is problematic both experimentally (e.g., awakenings during sleep studies) and from a welfare perspective (e.g., increasing stress) ^7^. Moreover, manual measurements at discrete times cannot detail *when* and at *what rate* animals eat—information which may be key to understanding changes in feeding behaviour upon genetic or procedural effects, as well as detection and intervention for welfare monitoring. Measuring continuous feeding alongside general activity traces enables discrimination between animal ‘food-intake’ (i.e., the mass of food consumed), ‘feeding-behaviour’ (i.e., time spent seeking food, yet not necessarily eating), and ‘activity’ (i.e., active movement, including but not limited to feeding-behaviour ^8– 11^).

Improved methods have been developed to automatically or semi-automatically monitor animal feeding and activity. There is an increasing interest in the automated monitoring of behaviour in the home cage, including multi-centre initiatives to promote such technology ( https://www.cost-teatime.org/about/technologies/). Examples include but are not limited to advanced whole-cage monitoring suites (e.g., Promethion Cages (Sable Systems International) ^12^ and CLAMS (Columbus Instruments International) ^13^), cage integrations (e.g., the Feeding Experimentation Device (FED3, NIH, RRID:SCR_015942) ^14^, passive infrared sensors (PIRs, RRID:SCR_022329) ^15^, and CageView ^16^), and video-based systems (e.g., PhenoTyper (Noldus Information Technology, RRID:SCR_024696) ^1^ and DeepLabCut (RRID:SCR_021391) ^17^). Video-based systems are improving; however, these only detect activity and interactions with food—not the amount eaten—and can struggle when views are obstructed by bedding or cage enrichment ^1, 17^. High-end systems are expensive and often require the purchase of entirely new caging systems ^12– 14, 16^. Others require animal training (e.g., lever-pressing, which may confound experiments involving cognitive processes) and introduce technical complications like pellet-jamming, crumbling, and incompatibility with recording setups like electroencephalography (EEG) and electromyography (EMG) ^7, 14, 16^. Moreover, systems requiring working for food may alter normal feeding behaviour ^18^. An optimal animal-food interface should not hinder parallel measurements, should not restrict food access, and should remain adaptable to a range of home-cage environments and experimental paradigms.

Here we describe a new animal home-cage monitoring system—the *SnackerTracker*. This device wirelessly records food-intake and contains a sensor which measures relative cage light conditions. It continuously saves information both locally to an SD card and remotely via the open-source Arduino Internet of Things (IoT) platform, which enables real-time remote data monitoring. It was designed to mimic the way in which food is normally presented to laboratory mice (i.e., from a food hopper above the cage floor to prevent food from soiling). Development involved chassis computer-aided design (CAD) and 3D printing, electronics assembly, construction, software production, and benchtop hardware and software testing before in vivo application. Major device elements include the frame (chassis), electronic circuitry, and both operational and analysis software. Components come to a total material cost of £120 per device (at the time of writing) and have been made available on open-access platforms, making the *SnackerTracker* a system which researchers can download, construct, and customise according to study requirements.

To test the ability of the *SnackerTracker* to measure food intake and feeding behaviour *in vivo*, here we investigate normal patterns of food intake and activity in both control mice (wild-type, C57BL/6J) and cryptochrome-deficient mice having disrupted circadian rhythms (i.e., cryptochrome 1 and 2 double-knockouts, *Cry1 ^-/-^,Cry2 ^-/-^*). Both genotypes were studied under controlled light-dark (LD) conditions and in constant darkness (DD). The circadian clock maintains regular patterns of physiology and behaviour, even in the absence of environmental cues (e.g., light-dark cycles), therefore control animals are expected to show rhythmic daily behavioural patterns under both LD and DD. Mice are nocturnal; they are active and feed during the dark phase with a peak at dark onset, then are inactive with brief bouts of spontaneous activity during the light phase ^19^. This pattern persists under constant conditions (e.g., DD) with a period of slightly less than 24h ^20^. By contrast, while cryptochrome-deficient animals exhibit normal rhythms in LD (due to light suppression of activity—termed ‘negative masking’), cryptochrome-deficient mice are arrhythmic under DD (irregular bouts of activity throughout a 24h period) ^21, 22^. The *SnackerTracker* should be able to detect this expected shift to behavioural arrhythmicity and characterise whether the amount or pattern of feeding also changes. 

Here we validate the *SnackerTracker* system by measuring feeding behaviour and food intake in both control and cryptochrome-deficient mice under both normal diurnal lighting environments (LD) and constant conditions (DD). Measuring food intake patterns provide novel insights into mechanisms underlying circadian and metabolic phenotypes, making the *SnackerTracker* a valuable tool for studying laboratory mouse welfare.

## Methods

### *SnackerTracker* development

**Figure 1.**
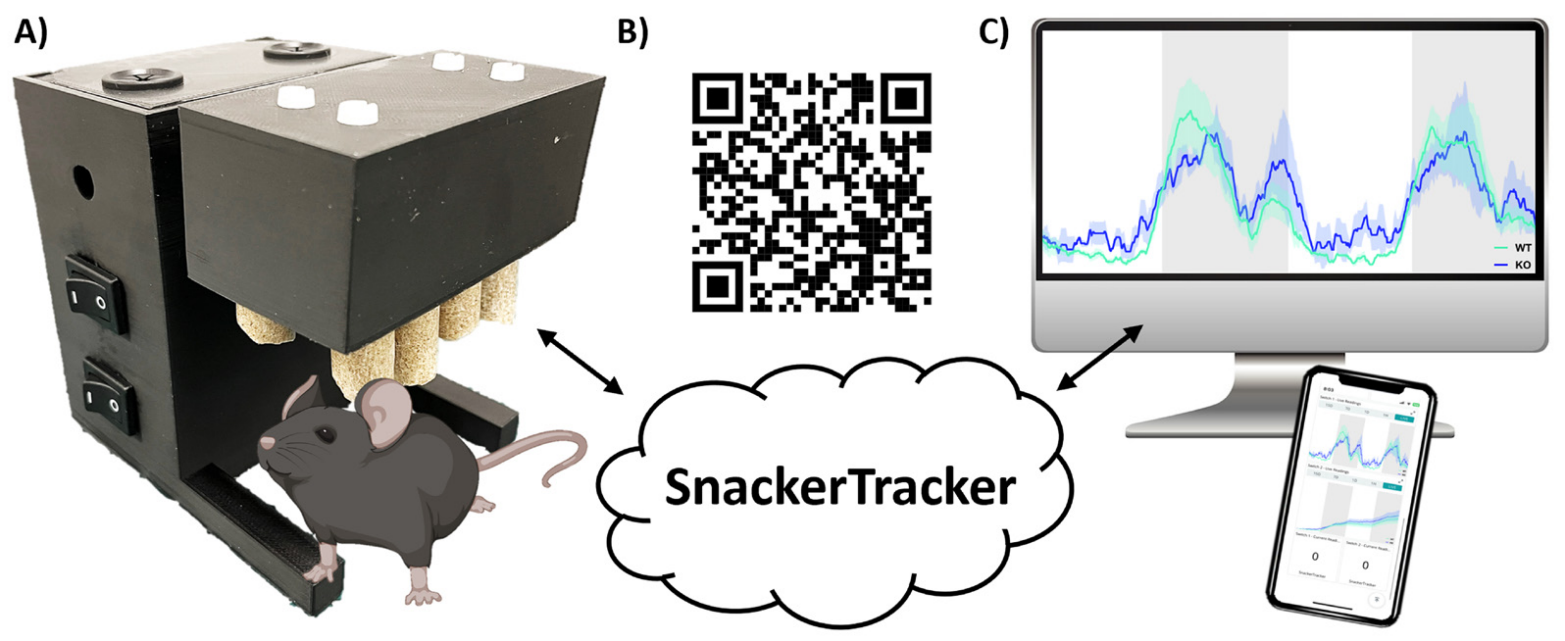
*SnackerTracker* design and operation. **A**) Shows the final prototype with a roughly-to-scale cartoon mouse. **B**) Provides a QR link to a *SnackerTracker* overview video ^24^. **C**) Illustrates data analysis, cloud-based data monitoring and device control capabilities.

***Overall design and chassis.*** The *SnackerTracker* was designed while keeping in mind the criteria and constraints described in ED - 1 ^23^. The final product and a summary of its development, construction, and application is provided in Figure 1 and illustrated in supplemental videos and material printouts ^24, 25^. The chassis was created using TinkerCAD computer-aided design (v2022, AutoDesk Inc., San Francisco, CA, USA), visualised using BabylonJS (v5.3.0, BabylonJS, San Diego, CA, USA), and animated using Maya (v2024.1, RRID:SCR_014287, AutoDesk Inc., San Francisco, CA, USA). MeshLab (v2022.02, RRID:SCR-003430, ISTI – CNR, Pisa, TUS, IT) fixed mesh errors by removing duplicate vertices, duplicate faces, and merging close vertices (10% merging distance). Healed meshes were exported as .stls without face colour, binary encoding, or materialised colour encoding. FreeCAD (v0.21.1, RRID:SCR_022535) was used to generate technical drawings (see ED - 2 ^23^). CAD objects were imported to Ultimaker Cura (v5.1.0, RRID:SCR_018898, Ultimaker BV, Utrecht, Netherlands), rendered (see ED - 3 ^23^ for specifications), then uploaded to an Ultimaker S3 (Ultimaker BV, Utrecht, Netherlands; RS Components, 192–5469) for 3D printing using 2.85mm diameter acrylonitrile butadiene styrene (ABS) black and polyvinyl alcohol (PVA) natural (Ultimaker BV, Utrecht, Netherlands; Farnell, 1621 and 9731 respectively). The glass printing plate was buffered with glue to ensure good print adhesion (UHU, Bühl, BW, DE; Viking, 3583323) and the print queued via USB (Lexar, San Jose, CA, USA; RS Components, 230–4402). Supports were removed carefully from the main chassis body, which was then buffered with acetone upon print completion (Thermo Scientific, Loughborough, LEICS; Fisher Scientific, 10666182). Detailed instructions, resources, and specifications are available open source via Figshare ^25, 26^, NIH 3DP ^27^, protocols.io ^28^, and the Arduino Project Hub ( https://projecthub.arduino.cc/marissamueller/snackertracker-d86bf3, also see Data and Software Availability), each of which elaborate upon the methods described below (including specific timeframes, potential sources of variability, and means of controlling bias).

***Electronics and mounting.*** Circuits were designed and schematically represented using CircuitDiagram (v2023, https://www.circuit-diagram.org/). Electronics were sourced (see ED - 4 ^23^ and Figshare ^25^ for the complete bill of materials), soldered using lead free 0.7mm-diameter solder wire (MultiComp Pro, London, LDN, UK; Farnell, 509–0600), then mounted to the chassis. 2 rows of 4-per-row 2.54mm pitch M-M pin headers (Molex, Lisle, IL, USA; RS Components, 90120–0761) were inserted into E+/E-/A-/A+ and GND/DT/SCK/VCC through-holes on a HX711 signal amplifier (Radwell, Newcastle-under-Lyme, NCL, UK; Radwell, 126663068) and soldered with load cell (LC) wires (Adafruit, New York, NY, USA; DigiKey, 4540) (red/E+, black/E-, white/A-, and green/A+). HX711 headers were soldered to the Arduino MKR MEM protoboard (Arduino, BCMI, Redmond, WA, USA; Mouser Electronics, ASX00008), with three 2.54mm pitch M-F pin headers (Harwin, Portsmouth, HA, UK; Mouser Electronics, M20-7821146) and a 10kΩ resistor (RS PRO, Corby, NTH, UK; RS Components, 707-8300). The protoboard underside was soldered to facilitate circuitry detailed in ED - 5 ^23^ using small u-shaped jumper wires as required (3M, Saint Paul, MN, USA; The Pi Hut, FIT0122). The MEM shield was stacked atop an Arduino MKR Wi-Fi 1010 microcontroller (Arduino, BCMI, Redmond, WA, USA; Mouser Electronics, ABX00023), which includes an on-chip real-time clock (RTC), such that MKR input/output (I/O) pins operate through MEM proxies. Four 0.2mm ^2^, brown 24 AWG, 7/0.2mm connective wires with PVC insulation (RS PRO, Corby, NTH, UK; RS Components, 196-4211) were cut to 10cm and 1cm was stripped at each end. One bare end was crimped to a vinyl-insulated female receptacle spade connector (6.3 × 0.8mm tab size) (RS PRO, Corby, NTH, UK; RS Components, 534-351), which in turn was joined to one of the two contacts on each of two on-off SPST 19.4 × 13mm panel mount toggle switches (E-SWITCH, Minneapolis, MI, USA; Farnell, RA1113112R). The other bare end was soldered to single M-M pin headers. Two more connective wires were similarly cut and stripped, yet with both ends soldered to single M-M pin headers. 3mm diameter (3:1) heat shrink wrap (RS PRO, Corby, NTH, UK; RS Components, 700-4532) was cut into 1cm segments and bonded around all wire-pinhead junctions. Two M-F pin headers were soldered to a 1in ^2^ accessory protoboard (SparkFun, Boulder, CO, USA; Mouser, PRT-08808) with each end of a light-depend-resistor (LDR) (LPRS, Witney, OX, UK; Farnell, N5AC501085) affixed in each M-pin through-hole. Bare lithium-polymer (Li-Po) battery (RS PRO, Corby, NTH, UK; RS Components, 144-9405) wires were crimped to 30–24 AWG JST PH female crimp terminals (JST, Doshomachi, Chuo-ku Osaka, Japan; RS Components, 820-1456) and inserted into JST PH housing (2mm pitch, 2 way, 1 row) (JST, Doshomachi, Chuo-ku Osaka, Japan; RS Components, 820-1466) such that, upon inserting into the Arduino MKR 1010’s JST-PH port, the black Li-Po wire was closest to (and red furthest from) the AREF pin header. All electrical connections were confirmed using a multimeter (DURATOOL, Taichung City, TW, CN; Farnell, D03047).

Cyanoacrylate glue (RS PRO, Corby, NTH, UK; RS Components, 473-461) was used to affix the following standoffs and nuts to the chassis for electronics mounting: four 10 + 5mm M2 M-F hex standoffs (Mac 8, Canoga Park, CA, USA; RS Components, 500-6621) to the chassis body wall; one 10mm (cut in half to 5mm) M2.5 F-F hex standoff (The Pi Hut, Haverhill, SF, UK; The Pi Hut, 104329) to the opposite chassis body wall above the LDR opening (pushed in, but not so far as to obstruct the vertical slot above the opening); two M5 hex nuts (7.8mm width, 4mm height) (RS PRO, Corby, NTH, UK; RS Components, 232-6976) to the interior chassis body bridge for eventual LC attachment; and four 6mm M4 standoffs (Essentra Components, Kidlington, OX, UK; RS Components, 102-6429) to the mount plate. The Arduino MKR 1010 was vertically mounted to the four M-F standoffs with M2 hex nuts (RS PRO, Corby, NTH, UK; RS Components, 483-0316) with the stacked MEM configuration. The LC was inserted through the chassis body and secured to the bridge using two trimmed 20mm M5 screws (MultiComp Pro, London, LDN, UK; Farnell, MP008089) and M5 washers (RS PRO, Corby, NTH, UK; RS Components, 232-6959) between the LC and bridge (two per screw). Strips of 20mm hook and loop Velcro tape (RS PRO, Corby, NTH, UK; RS Components, 890-4161 and 890-4168 respectively) were trimmed to 3cm-long segments and adhered to the base of the chassis interior and Li-Po battery. This secured the battery such that wire leads emerged nearest (and directly underneath) the Arduino MKR 1010’s JST port. An 18 × 18mm glass cover slide (Scientific Laboratory Supplies, Nottingham, NTT, UK; Scientific Laboratory Supplies, MIC2162) was placed into the vertical slot above the LDR opening in the chassis wall.

The LDR-mounted protoboard was then attached to the chassis interior, with the sensor visible from the exterior through the glass cover, using trimmed 10mm M2.5 screws (TR Fastenings, Uckfield, SX, UK; Farnell, TRNSE-1207-M2.5-10). Toggle switches and wires were pushed through each of the two rectangular through-holes from the outside-in below the LDR. One M-pin lead extending from each switch was connected to an F-pin header soldered nearest the HX711 on the Arduino MKR MEM protoboard. The other lead from the top toggle switch was plugged into pin 7 on the Arduino MKR MEM shield, and from the bottom toggle switch to pin 6. One end from each of the two remaining wires (having M-pins at both extremities) was plugged into F-pin headers on the LDR-mounted protoboard. The opposite end of one was plugged into the third F-pin header on the Arduino MKR MEM protoboard, and the other into the Arduino MKR MEM’s DAC0/A0 pin. Two trimmed 10mm M4 screws (TR Fastenings, Uckfield, SX, UK; Farnell, TRNSE-1207-M4-10) were inserted through the bottom LC holes protruding from the chassis and affixed to the hex nuts along the midline of the mount plate.

When ready for use, food pellets were pressed through the food holder (inside-out) with tape affixed to the interior to secure the tip of each pellet to the chassis (Thorlabs, Newton, NJ, USA; Decker Tape Products, T743-1.0). The food holder was affixed to the mount plate with four M4 screws (the same as used for LC attachment). Two 15.9mm diameter cable retention grommets (Essentra Components, Kidlington, OX, UK; Farnell, PGSD-2) were pushed into the case cover, which was then inserted into the chassis body after plugging the Li-Po battery into the Arduino MKR 1010’s JST port. A U3 micro-SDXC card (Integral, Bristol, BS, UK; Farnell, INMSDX64G10-90U1) was inserted into the Arduino MKR MEM shield after being re-formatted from exFAT to FAT-32 format using MiniTool Partition Wizard (v12.7, MiniTool, Vancouver BC, Canada) for system compatibility. Videos detailing *SnackerTracker* design and construction (in the same order described here) are provided open-source via Figshare ^24^, with additional protocols, timelines, sources of variability, error-control measures, and resources available as mentioned previously at NIH 3DP ^27^, protocols.io ^28^, and the Arduino Project Hub (see Data and Software Availability).

***Software and operation.*** Operational software ^29, 30^ was written using the Arduino Editor and Integrated Development Environment (IDE) (v2.0, RRID:SCR_024884, Arduino BCMI, Redmond, WA, USA). The script includes device calibration as a mandatory part of *SnackerTracker* initialisation, which first involves removing the case cover and ensuring that a charged Li-Po battery is connected to the JST port. Note that USB-Li-Po power-switching occurs automatically; the USB port powers the device and charges the Li-Po battery when connected to an external outlet, and the Li-Po-battery powers the device in the absence of USB connection. Li-Po batteries can therefore either be charged externally to the *SnackerTracker* using JST charge adapters (DFROBOT, Kington, HEREF, UK; Farnell, DFR0668), or within the *SnackerTracker* if plugged into the JST port, with the microchip connected to another power supply via USB. The food holder should be empty and placed on the mount plate. The weight of pellets which are to be added should be obtained and recorded using a scale with 0.01–0.1g precision (DURATOOL, Taichung City, TW, CN; Farnell, D03409). After opening the Arduino Editor script and running the Arduino Create Agent—a small background application which enables communication between programming computers and Arduino boards—code can be uploaded via male USB A to male micro USB B cable (RS PRO, Corby, NTH, UK; RS Components, 182-8872). The script contains a setup() function for device initialisation, and a loop() function for data recording. setup() first connects to the HX711 and RTC, then prompts the user to enter the mass of food which is to be added in the Editor’s serial monitor. After providing this calibration mass, the user has 3 minutes to add taped pellets to the food holder to then screw to the mount plate. This 3-minute timeframe can be adjusted within the script if needed. setup() then tares and calibrates the device, connects to on-board LED lights, checks that an SD card is present, creates an output .csv file on the SD card, then passes control to loop() for data recording. The USB cable can be disconnected for wireless device operation, or can thereafter be connected to an external power supply via USB cable connection to a USB port (MultiComp Pro, London, LDN, UK; Farnell, 2843448) and wall adapter according to the country of use (e.g., HAMA, Monheim, ME, DE; Farnell, 73014150) to enable longer recordings exceeding the maximum Li-Po battery life of ~48h. Irrespective of power supply, loop() reads and writes input load cell, LDR, and RTC data to the output .csv file. The program continually checks that the SD card is present and indicates connectivity or data logging errors flashing the red on-board LED light. An Arduino Internet of Things (IoT) platform facilitates Wi-Fi connection and real-time data monitoring from any networked system, including via the IoT Remote App for Apple or Android device (v1.6.0). Loop() repeats and records data at the script-defined frequency (e.g., 1Hz, which can be adjusted) until components are disconnected or power is lost (e.g., disconnecting both the USB and Li-Po battery). The SD card can then be removed for data download using a microSD adapter (EATON TRIPP LITE, Woodridge, IL, USA; Farnell, U452-000-SD-A) and subsequent processing. Analysis code was written in MATLAB (vR2017b, RRID:SCR_001622, The MathWorks Inc., Natick, MA, USA). Again, further information regarding *SnackerTracker* software and its use is referenced in Data and Software Availability.

### Validation tests

Preliminary validation tests assessed 1) device calibration accuracy, 2) drift, 3) time-sensitivity, 4) LDR function, 5) combined operation, and 6) correlation to manual measurements of daily food intake upon *in vivo* application:

1) Calibration testing involved initialising *SnackerTrackers* using pellet-masses roughly equivalent to that of a i) empty (28g), ii) half-full (55g), and iii) full (82g) food holder. After initialisation, pellets were sequentially removed and manually weighed for comparison to *SnackerTracker* recordings. This assessed both the accuracy of *SnackerTracker* measurements and whether this accuracy depends on the calibration mass selected (which it should not).

2) Drift is a phenomenon wherein device performance deteriorates after calibration. This presents as erroneously fluctuating measurements over time ^31^. To evaluate drift, a known mass was placed on the food holder. Data was collected for eight hours without interference to determine whether measurements ideally remain constant or incorrectly shift over time.

3) Time-sensitivity tests assessed the accuracy of *SnackerTracker* timestamps compared to manual recordings. The *SnackerTracker’s* Arduino MKR 1010 microchip features two means by which time can be registered in operational code: via script-based frequency loops, or by extracting information directly from the on-chip RTC. Loop() records data at a user-defined frequency (e.g., 1Hz), however hardware and processor burden may cause software lag. This can result in mass/light measurements being erroneously reported as occurring earlier in time. Alternatively, RTCs register time independent of peripheral mass/light measurements and should prevent lag. To test this and confirm use of the RTC over looped LC timestamps, *SnackerTracker* data was collected, using both RTC and LC recordings, during the sequential removal of 12 food pellets (one every 30 minutes). Each pellet was manually weighed, and removal times were documented for comparison to *SnackerTracker* LC/RTC data.

4) LDR recordings are intended to confirm scheduled light-dark cycles, which is important when assessing or controlling for circadian and homeostatic functions (e.g., sleep). To test LDR function, a researcher first lowered an opaque box overtop the *SnackerTracker*, then then repeatedly flipped the room’s light switch to test the ability of the *SnackerTracker* to identify ‘ramping’ and absolute periods of lights being ‘on’ or ‘off’, respectively.

5) The combined test simulated cage light conditions (i.e., turning lights ‘on’ and ‘off’), animal food consumption over time (i.e., manually removing pellets from the food holder), and switch functionality (as switches can be used for event-marking and data-filtering during post-processing). Lights were scheduled at two cycles of 30s ‘off’ and 60s ‘on’, followed by a final 30s ‘off’. Pellets were removed during the light-phase, and switches 1 (top) and 2 (bottom) were flipped directly before lights turned ‘on’ or ‘off’, respectively. This was repeated with and without IoT connectivity. The test mimicked what would be expected *in vivo* (with pellet removal occurring in the dark phase for nocturnal animals) and evaluated *SnackerTracker* function before exposing the device to animals.

6) *SnackerTrackers* were tested *in vivo* to assess correlation to manual measurements (n=24). Devices were loaded with food pellets, weighed, placed in animal cages for 24h, then weighed again. The calculated difference between the mass of the *SnackerTracker* with food before and after this 24h period (i.e., measuring daily food intake as equivalent to this mass difference) was recorded and compared to values returned by the *SnackerTracker* itself. This assessed *in vivo* measurement accuracy. Then, animal food intake was measured over a 24h period by the traditional manual method using the same mice (i.e., weighing pellets, adding them to each cage, then removing and weighing the pellets remaining after 24h timeframe either before or after the *SnackerTracker* recording). Manual measurements vs *SnackerTracker* measurements were compared to evaluate whether the method of food presentation altered daily food intake, with all comparisons being paired (i.e., from the same mouse). Using a separate cohort subset housed with fresh alpha-dry bedding in standard open-top cages (n=24), *SnackerTracker* recordings were obtained for 47–92h such that bedding could be sieved thereafter to isolate any crumbs which would have been removed from the *SnackerTracker* but not eaten. 5 datasets were excluded where recordings terminated prematurely (i.e., due to battery failure), yielding a final sample size of n=19. Crumb masses were documented and normalized by recording time to determine the average amount of crumbling per day.

### Animal breeding and husbandry

All animals were housed in Digital Ventilated Cages ^®^ (DVCs) (Tecniplast, Buguggiate, VA, Italy) ^32^ with individual LED lighting (Leddy, Tecniplast, Buguggiate, VA, Italy) at the University of Oxford’s Biological Sciences Building with *ad libitum* access to food and water. Facilities maintained specific pathogen free conditions, where serology, parasitology, and bacteriology reports were acquired from Surrey Diagnostics Ltd., Cranleigh, SRY, UK. During all experiments, the only reported positives upon health screening were 2/7 for Enteromonas sp. and 2/7 for Tetratrichomonas sp. No animals in this study had a history of previous regulated procedures ( *a priori* exclusion criteria). Both adult males and females were tested as single-animal experimental units, with the cohort of n=12 mice (all C57BL/6J background) consisting of n=3 control males, n=3 control females, n=3 cryptochrome-deficient ^22^ males, and n=3 cryptochrome-deficient females aged (mean $\bar{x}$ ± standard error *σ _M_*, in weeks) 21.6±2.6 for control and 24.5±4.1 for cryptochrome-deficient mice. Control and cryptochrome-deficient genotypes were confirmed by Transnetyx PCR (Cordova, TN, USA).

### Experiment setup for comparison of control versus cryptochrome-deficient mice

**Figure 2.**
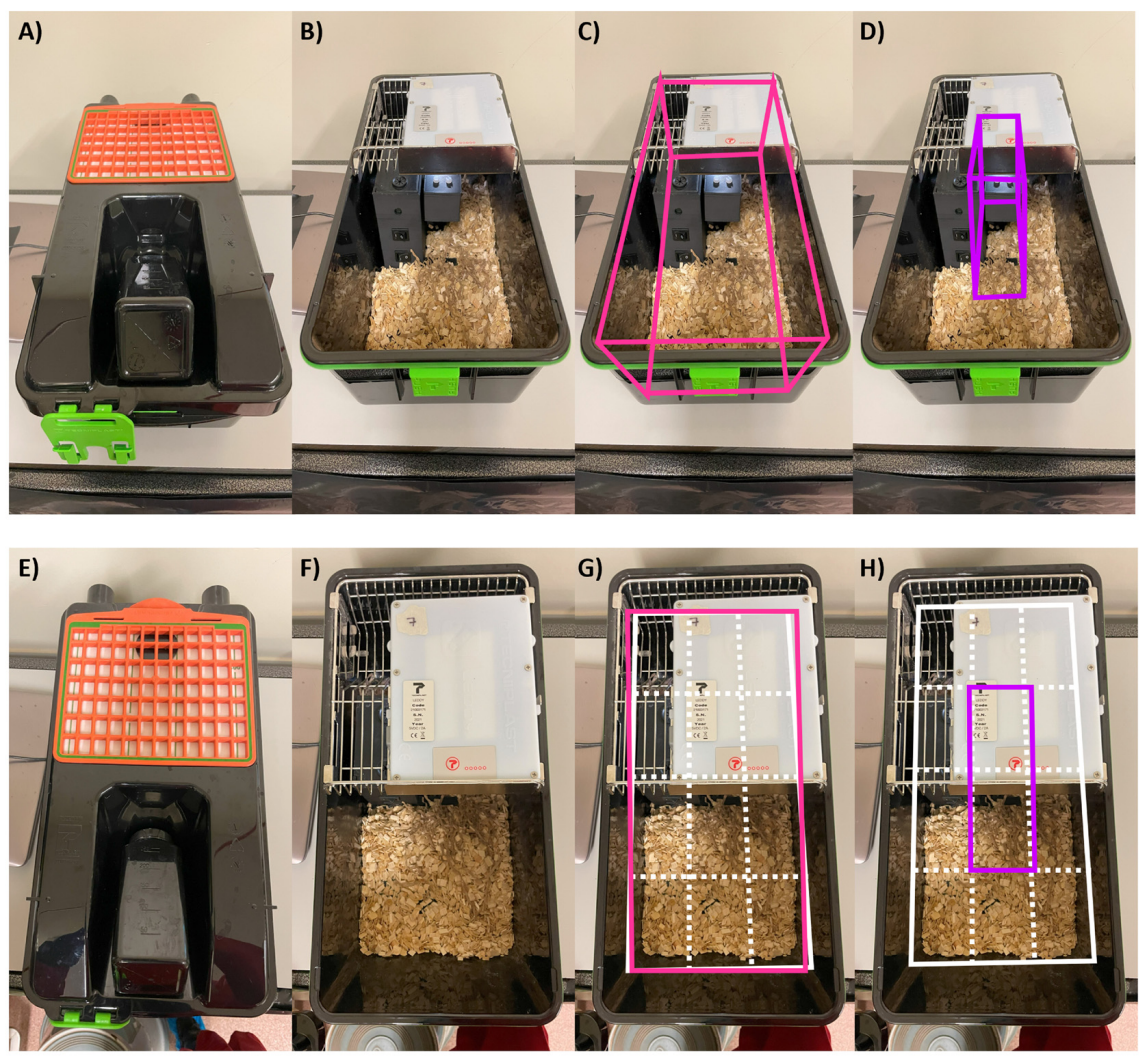
*SnackerTracker* setup in activity-tracking DVCs. Opaque LED-equipped cages are shown **A**, **E**) with and **B**– **D**, **F**– **H**) without the lid. Areas which are considered for DVC-based activity monitoring are illustrated for **C**, **G**) ‘whole-cage’ measurements (pink, encompassing all areas) and **D**, **H**) ‘mid-cage’ measurements (purple, the area only around the *SnackerTracker*).

Battery-operated *SnackerTrackers* were introduced to a total of n=12 mice singly housed in Leddy-equipped DVCs ^32^. Following at least one day of habituation, 2×48-hour recordings were collected per animal: the first under a 12:12 h light-dark (LD) cycle, and the second in constant darkness (DD). DVCs recorded animal activity both for the ‘total’ cage (i.e., activity in any area) and for the ‘central’ region (i.e., solely around the *SnackerTracker*) for comparison to *SnackerTracker* data (see Figure 2). The individuals conducting *in vivo* experiments were not blind to animal genotype, sex, or lighting condition. Manual measurements were recorded as the difference between the mass of the *SnackerTracker* with food before and after recording onset and termination. Potential confounders such as cage location and environmental conditions were controlled for by ensuring all animals were placed within the same rack on the same wall of the housing room in practically identical DVCs (controlled lighting, humidity, and temperature) ^33^. Recordings commenced and were terminated at the same time for all mice, occurring after technician inspections to minimise physical disturbances and otherwise random day-to-day fluctuations (e.g., sound).

### Statistics

Power calculations were conducted using G*Power (v3.1.9.7, RRID:SCR_013726) ^34^ ( *a priori* unless otherwise stated), and further statistical analyses using GraphPad Prism (v10.2, RRID:SCR_002798, Dotmatics Ltd., Bishop's Stortford, HRT, UK). T-values are reported as t(df) for all t-tests (two-tailed), and F-values as F(DFn,DFd) for linear regression and ANOVA. Significance is defined as p<α=0.05 throughout. All data are reported as $\bar{x}$ ± *σ _M_* and, where applicable, were analysed blind to genotype/condition.

Coefficients of determination (r ^2^) evaluate the spread of data following simple linear regression for calibration tests, the step test, and preliminary *in vivo* tests of *SnackerTracker* correlation to manually obtained masses and typical cage-feeding. Regression employs the least-squares model with no special handling of outliers, under the assumption that underlying data follows a Gaussian distribution and is constrained at Y=0 (confirmed by QQ plots and Shapiro-Wilk normality tests). Correlation significance is tested against a null hypothesis of X=Y (two-tailed), with all cohorts exceeding the required sample size of n=9 (β=α=0.05) to detect effect sizes of f ^2^=2.0 (estimated via pilot testing). Pearson’s r correlation matrices compare *SnackerTracker* vs DVC outputs (i.e., *SnackerTracker* feeding, *SnackerTracker* animal-device interactions, whole-cage DVC recordings, and mid-cage DVC recordings).

Paired two-tailed t-tests evaluate differences in animal body mass data before and after *SnackerTracker* recordings (exceeding the sample size of n=7 required to detect a 5% change in body mass at β=α=0.05, with $\bar{d}$ =0.9 and *σ *_M_** =0.5 as determined from pilot data). The same test was conducted to compare manual vs *SnackerTracker* daily feeding and activity (yet this time *post hoc,* which at the most conservative estimate yielded a power of β-1 = 0.803 for α=0.05 and n=12 combining male and female data). These metrics were not suitably powered to detect sex differences (β-1 = 0.803 for α=0.05 and n=6). Unpaired two-tailed t-tests assess overall effects of genotype on animal body mass, food intake, and feeding behaviour under assumptions of normality and equal variance (confirmed by QQ and homoscedasticity plots, respectively).

48-hour recordings are cropped to 24-hour timelines and shown as Zeitgeber time (ZT) for LD or circadian time (CT) for DD. Custom MATLAB scripts ^29, 30^ fit sine curves to each individual timeline using the Curve Fitting toolbox (v3.8, RRID:SCR_001622, The MathWorks Inc., Natick, MA, USA) and Sum of Sine regression model. This employs the non-linear least-squares method using the Trust-Region algorithm and default constraints to generate a curve according to $\sum_{i=1}^{j}a_{i}\sin(b_{i}x+c_{i})$ . For single-term models (j=1), extracted parameters of interest include waveform displacement ( *d* = *a _i_*), angular frequency ( *ω* = *b _i_*), and goodness of fit (r ^2^). The custom MATLAB script transforms amplitude and frequency data to return curve intensity (amplitude, *A* = 2 × *d*) and period ( *T* = $\frac{1}{f}$ = $\frac{2\pi }{\omega }$ ).

3-way RM-ANOVA returns overall and interactive effects of light condition (LD vs DD), genotype (control vs cryptochrome-deficient mice), and sex (male vs female) on animal feeding and food-seeking behaviour matched by light condition (assuming sphericity and confirmed by residual plots). This applies to overall values across 24h periods, isolated light-phase and dark-phase data for LD conditions and extracted periodicity and intensity metrics for fitted curves. *Post hoc* calculations determined that the resulting data was only ~45% powered to detect a large effect size of f=0.4 at a significance level of α=0.05.

No data was excluded from any of the validation tests presented. Data points for *in vivo* experiments were excluded and repeated if *SnackerTracker* recordings were terminated early due to battery failure or experimenter error (resulting in 24 excluded datasets), if *SnackerTracker* data failed to record to the microSD card (resulting in 2 excluded datasets), or if feeding traces exhibited significant chipping artifacts preventing pre-processing software from deriving timeline/summary metrics (resulting in 5 excluded datasets). No data was excluded after pre-processing. All criteria were determined *a priori*.

## Results

### *SnackerTracker* design and outcomes

**Figure 3.**
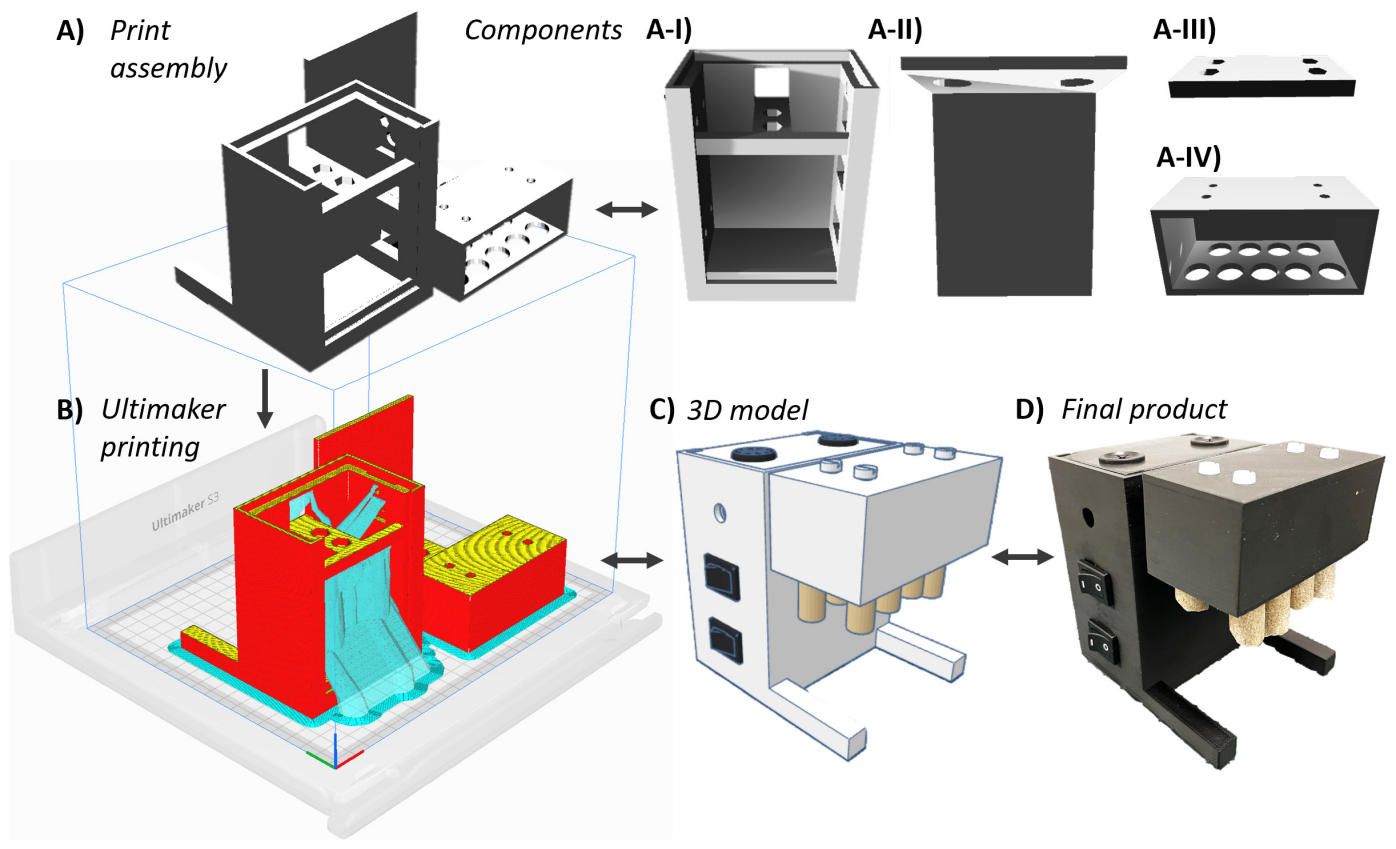
*SnackerTracker* chassis 3D modelling and materialisation. **A**) CAD-generated chassis component models were rendered into a print configuration consisting of four individual parts: the I = main body, II = case cover, III = mount plate, and IV = food holder. **B**) Shows the print configuration imported into Ultimaker Cura printing software (red = outermost chassis wall; yellow = top surface; and cyan = removable supports). This process enabled the transformation of the **C**) original 3D *SnackerTracker* TinkerCAD model into **D**) the final assembled device. See Figshare for videos showing 3D printing and construction (Data and Software Availability).

***Chassis.*** 11 prototype iterations preceded the present design. The chassis consists of four parts: I = main body, II = case cover, III = mount plate, and IV = food holder. Schematics are provided for each in ED - 2 ^23^, with further information available in Data and Software Availability (including open-source provisions via NIH 3DP ^27^, protocols.io ^28^, Figshare ^26^, and the Arduino Project Hub). Figure 3 illustrates chassis development progression from *SnackerTracker* CAD modelling through component isolation, print assembly, and the corresponding final product ^27^.

**Figure 4.**
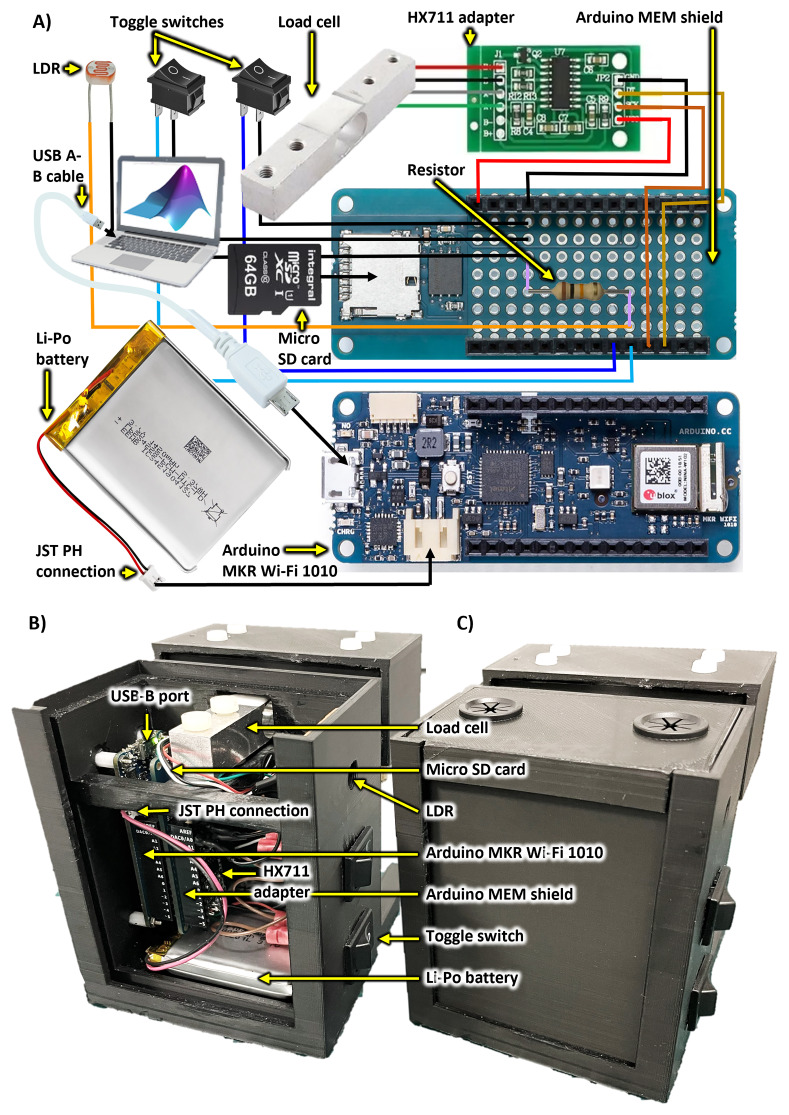
*SnackerTracker* electronics and key components. Labelled diagrams are provided both **A**) as a schematic representation and **B**– **C**) mounted within the chassis both **B**) without and **C**) with the case cover. Refer to ED - 4 ^23^ and ED - 5 ^23^ for component specifications and circuit schematics, respectively, and to Figshare for construction videos ^24^.

***Electronics.*** Figure 4 shows electronics pictorially and as mounted to the chassis. Schematics are provided in ED - 5 ^23^. See Data and Software Availability for links to additional electronics configuration details and resources (e.g., protocols.io ^28^, the Arduino Project Hub, and NIH 3DP ^27^).

***Software and analysis.*** Operational software and analysis scripts are available via the Arduino Project Hub, Zenodo ^29^, GitHub ( https://github.com/marhmue/SnackerTracker-Code---Core), and Figshare ^30^ (also see Data and Software Availability). The user interface is shown in ED - 6 ^23^. Analysis code requires the user to provide information described in ED - 7 ^23^ for data import and nine filtering steps, which are described in GitHub and ED - 8 ^23^. Steps include: 1) raw data visualisation and switch assessment; 2) crop specifications; 3) mass filtering; 4) plateau filter application; 5) interactions assessment and frequency analysis (i.e., deriving cumulative food intake and animal-device interactions to obtain rates of feeding and food-seeking behaviour over time); 6) LDR filtering; 7) output summary; 8) cropping and alignment; and 9) data compression. Sample intermediate plots for each step are provided in ED - 9 ^23^. These are generated and saved for every processed *SnackerTracker* file to both visualise parameter effects and to thoroughly document filter application. A separate metadata .csv file is saved alongside the .csv containing raw and processed data. Another file is generated if data compression is specified (for a maximum of three total output .csvs). Sample output .csvs are curated for illustration in ED - 10 ^23^. Full files are provided and can be imported to other programs (e.g., GraphPad Prism) for graphical and statistical analysis.

### Testing and validation

**Figure 5.**
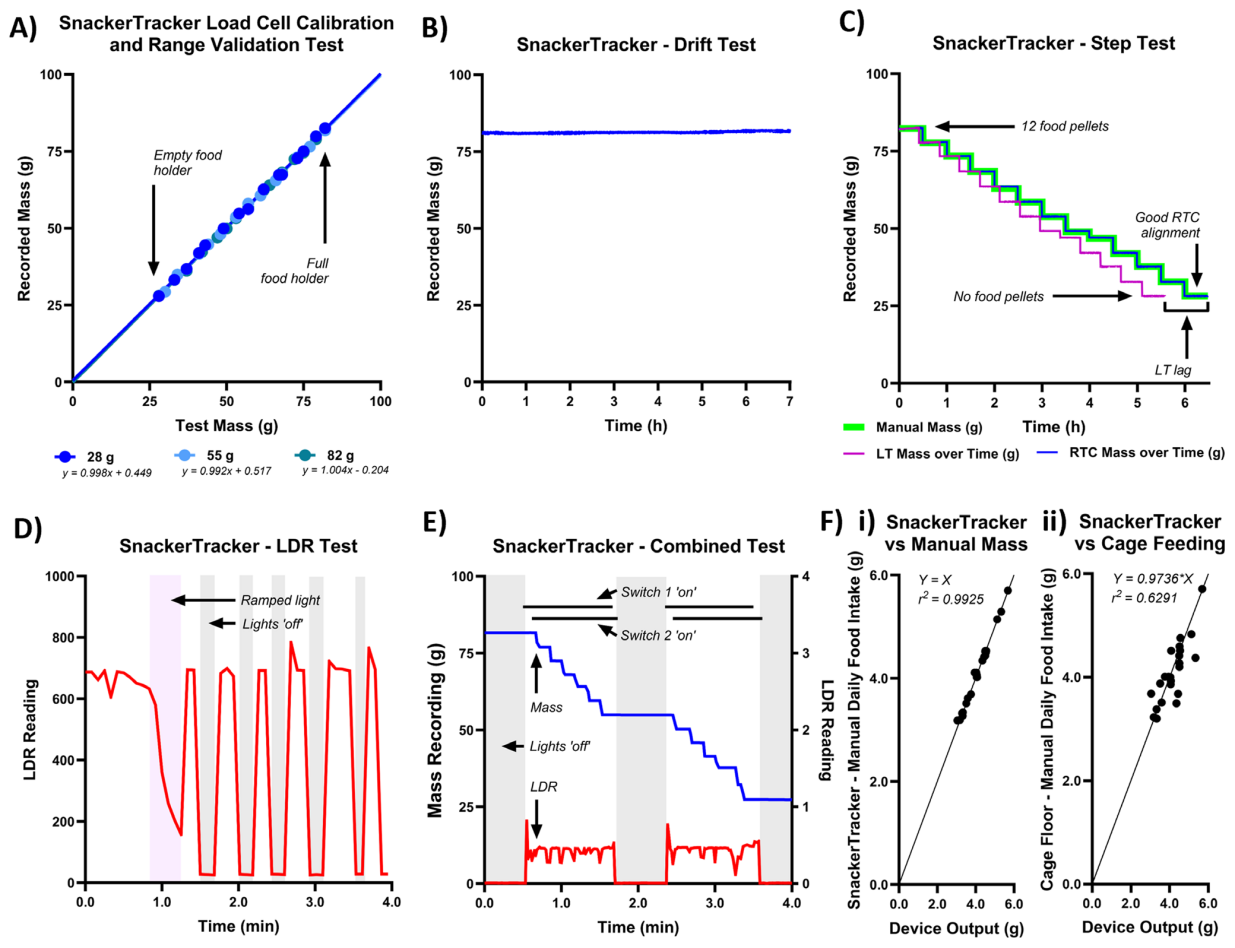
Results of *SnackerTracker* benchtop validations and preliminary *in vivo* testing. **A**) The load cell (LC) calibration and range validation tests show near-perfect agreement between *SnackerTracker* recordings and manually obtained measurements, regardless as to which calibration mass was used (here tested at 28g, 55g, and 82g corresponding to a food holder which is near-empty, half-full, and full respectively). **B**) The drift test shows insignificant measurement fluctuation when subjected to a load for 7h without further perturbation, which indicates good system performance. **C**) The step test demonstrates close agreement between manually obtained expected mass-time values and those resulting with use of the on-chip real-time clock (RTC). LC timestamps appear inaccurate and temporally compressed, therefore indicating the RTC as the superior time-measurement method. **D**) The light-dependent resistor (LDR) test shows that the *SnackerTracker* can both report dimming light and absolute periods of lights being ‘on’ or ‘off’. **E**) The combined test indicates that all outputs function as expected. Switch data is accurately returned to reflect switches 1 and 2 changing status directly before and after each time the lights are turned ‘on’ or ‘off. Reported masses align with 6 pellets being sequentially removed during each light phase, during which LDR recordings increase as expected. **F**) *SnackerTracker* data correlates **i**) very closely with the manually-weighed difference in the mass of the device with food before and after a 24h period, indicating good *in vivo* measurement accuracy; and **ii**) moderately with manual measurements obtained without the *SnackerTracker* during an independent 24h period in the same mouse cage (i.e., paired *SnackerTracker* vs non *-SnackerTracker* daily food intake, where manual data was obtained the traditional way by simply adding, removing, and weighing pellets). Daily food intake does not consistently increase or decrease when pellets are suspended in the *SnackerTracker* as opposed to traditional placement in the home cage.

**Figure 6.**
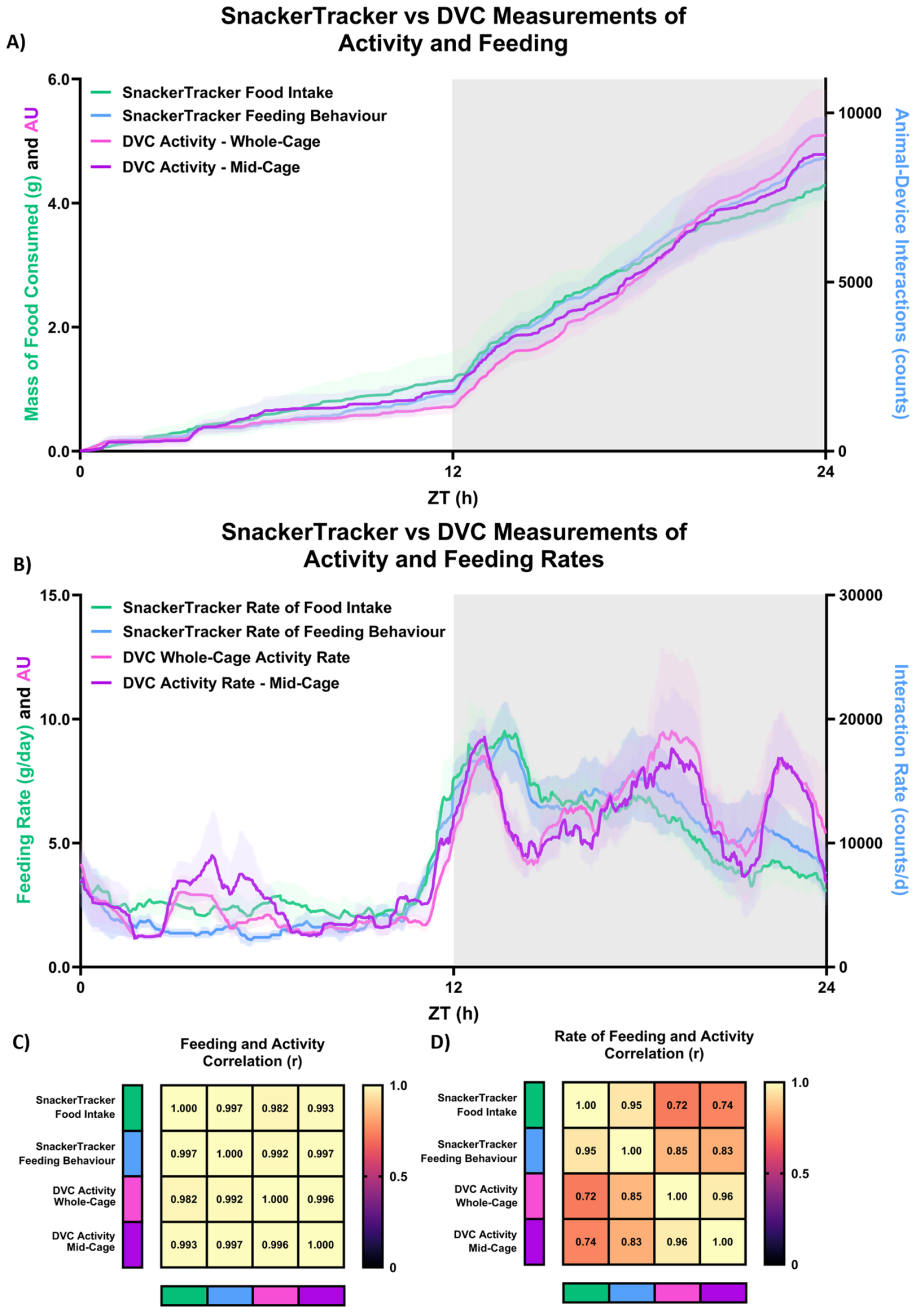
Correlations and agreement between *SnackerTracker* food intake, *SnackerTracker* feeding behaviour, and DVC activity recordings. Data from DVCs were collected across the entire cage, however recordings analysed were obtained solely from the middle-region where the *SnackerTracker* was located (refer to Figure 2). Daily traces for control (wild-type, C57BL/6J) animals housed under 12:12h light:dark conditions are shown both as **A**) cumulative food intake or activity counts, and **B**) as corresponding derivative rates. Pearson’s r correlation matrices assess agreement between the time-course of each recording method for **C**) cumulative and **D**) derivative plots corresponding to **A**) and **B**), respectively. Trendlines and shaded regions represent $\bar{x}$ ± *σ _M_*.

Results from the six preliminary validation tests are shown in Figure 5, with good correlations to DVC recordings (Pearson’s 0.992<r<0.997) illustrated in Figure 6. Collectively, these tests support *SnackerTracker* measurement validity. Outcomes of validation tests ( Figure 5) are as follows:

1) The calibration test ( Figure 5A) shows good alignment between *SnackerTracker*-recorded masses and known values, irrespective of the initial mass used for calibration. That is, expected masses correlate well with *SnackerTracker*-obtained values and corresponding trendline slopes do not differ significantly from an expected value of 1 (i.e., y = x) across all calibration masses (r ^2^=0.9987, F(1,14)=3.286, p=0.0913 for 28g; r ^2^=0.9987, F(1,14)=0.01674, p=0.9990 for 55g; and r ^2^=0.9996, F(1,14)=0.05310, p=0.8211 for 82g). This indicates that the *SnackerTracker* precisely returns the mass expected irrespective of the calibration mass selected.

2) No notable deviance from the initial values or random fluctuation was observed in the drift test ( Figure 5B). This suggests that simply performing regular device calibrations should suffice for reliable device performance (e.g., at the start of each *SnackerTracker* recording). Mean random fluctuations were functionally negligible (±0.045g) and informed the value to which the band-pass filter ( *bufferHere*) should be initialised (i.e., 2×0.045≈0.1g, see ED - 7 ^23^).

3) In the step test ( Figure 5C), the *SnackerTracker* recordings closely matched those of known mass values upon sequential load removal (r ^2^=0.9999). However, while masses plotted against RTC-time precisely reflected the manually documented times at which food was removed (final time difference≈2s), LC-time differed notably (time difference≈54min). This shows that frequency-based timestamps do not reliably correlate with real-time. Operational code was modified solely to extract RTC timestamps.

4) In the LDR test ( Figure 5D), both lights ‘dimming’ and absolute periods of lights being ‘on’ and ‘off’ can be identified in *SnackerTracker* outputs.

5) In the combined test ( Figure 5E), and as expected, mass measurements decreased during the light-phase with pellet removal. LDR recordings reflected light-status in the room, and switches are accurately shown as flipping either just before (switch 1) or after (switch 2) a change in lighting. When outside the recording room, the researcher successfully connected to the Arduino IoT and monitored recordings both from a networked laptop and mobile phone.

6) Then progressing to *in vivo* testing, Figure 5Fi shows very high correlation between *SnackerTracker* daily food intake (X) and the manually-obtained mass of the *SnackerTracker* (including food, Y) before and after 24h in a mouse cage (not differing significantly from a direct 1:1 relationship; Y=1.000X vs Y=X, r ^2^=0.9925, F(1,23)=0.004299, p=0.9483). This indicates good device accuracy *in vivo*. Figure 5Fii shows data comparing *SnackerTracker* outputs (Y) to manual mass recordings from cage-feeding (Y) from the same mouse over independent 24h periods. These do not significantly differ from an ideal direct relationship and demonstrate moderate correlation (i.e., Y=0.9736X vs Y=X, r ^2^=0.6291, F(1,23)=2.158, p=0.1554), suggesting that the method of food presentation does not consistently alter animal food intake in wild-type animals (i.e., in the *SnackerTracker* food holder as opposed to from the cage food-hopper or cage floor, notwithstanding likely error due to practical difficulties in recovering food manually). In the separate animal cohort (n=19), daily crumbling a manually measured at $\bar{x}$ =0.219±0.016g/d.

### Comparison of control versus cryptochrome-deficient mice

Control mice weighed significantly more than cryptochrome-deficient mice (27.3±1.82g vs 22.7±1.2g, t(10)=2.317, p=0.043), and *the use of SnackerTracker* did not lead to any changes in body mass after recordings (i.e., the mice did not show adverse weight loss; t(10)=1.179, p=0.266, which was furthermore not significant when assessing interactions by genotype (F(1,8)=0.02381, p=0.8812) or sex (F(1,8)=0.2045, p=0.6631).

**Figure 7.**
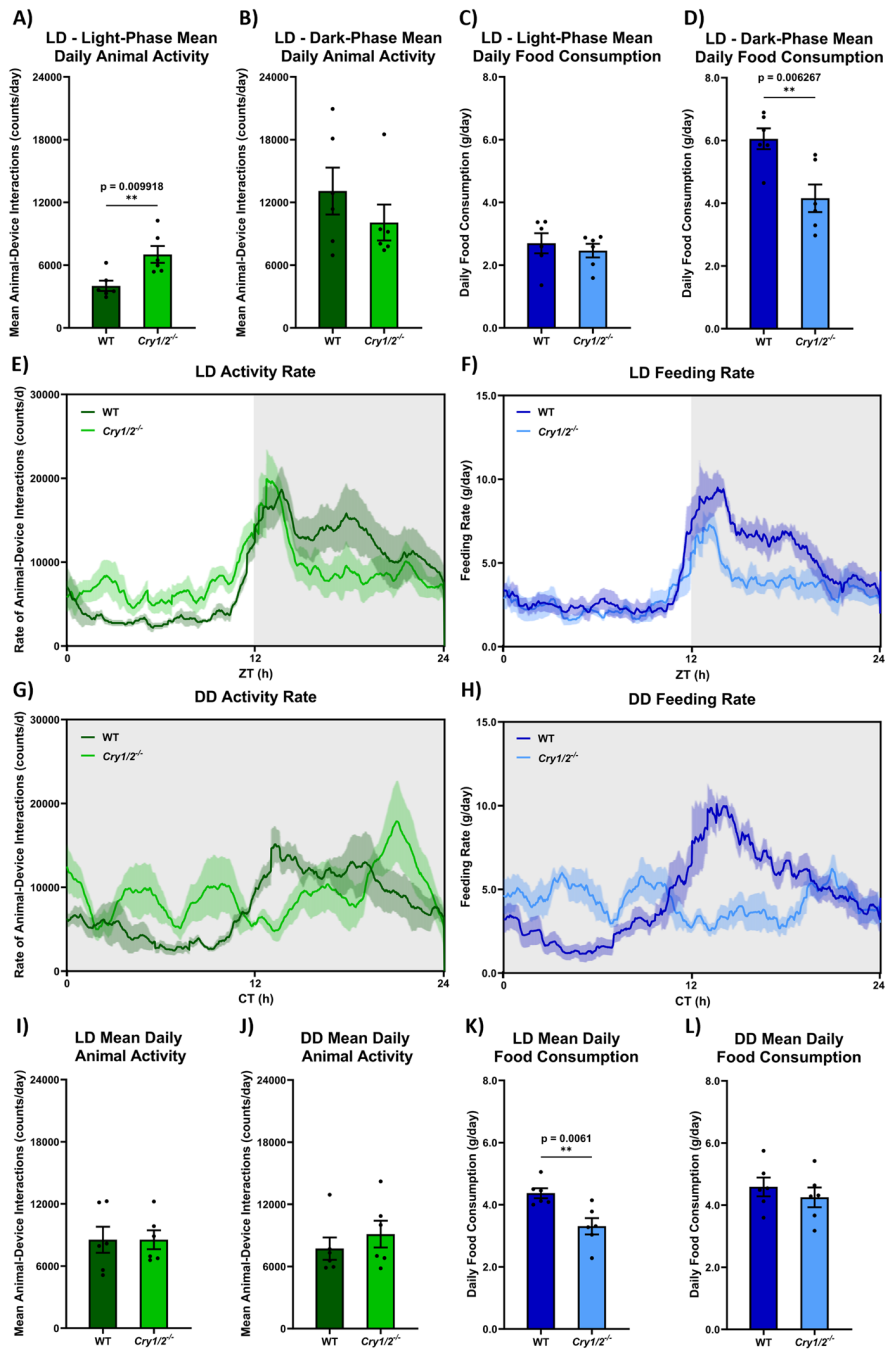
Control and cryptochrome-deficient animal activity and food intake differs across home-cage lighting conditions. When housed under a 12:12 light:dark cycle (LD), **A**) cryptochrome-deficient animals ( *Cry1 ^-/-^,Cry2 ^-/-^*) appear to be more active in the 12h light phase with **B**) no significant difference in the 12h dark phase compared to control animals (wild-type, C57BL/6J). However, **C**) the 12h of feeding in the light phase does not differ notably between control and cryptochrome-deficient animals while **D**) cryptochrome-deficient mice appear to eat less during the 12h dark phase. Corresponding 24h LD timelines of animal **E**) activity and **F**) food intake illustrate these differences within an overall expected nocturnal pattern of decreased light phase activity/feeding and increased dark phase activity/feeding. This diurnality is not ubiquitously conserved in DD; for both **G**) activity and **H**) feeding, control animals exhibit a similar pattern as in **E**– **F**), however cryptochrome-deficient animals lack this circadian behaviour and exhibit shorter bouts of activity and feeding bursts throughout the 24h timeline. While the *pattern* of activity clearly differs, 24h mean values for **I**) LD activity (corresponding to **E**) and **J**) DD activity (corresponding to **G**) shows no notable differences between *total* control and cryptochrome-deficient animal behaviour. Conversely, **K**) cryptochrome-deficient animals consume less food than controls over the 24h day in LD (corresponding to **F**), while **I**) overall food intake does not differ between genotypes in DD (i.e., cryptochrome-deficient animals then eat more, resembling the food intake of control animals). Male and female data are combined as $\bar{x}$ ± *σ _M_* due to no notable sex difference (p>0.05 for all measures). Sex-disaggregated data is provided in ED - 11 ^23^.

Figure 7 compares *SnackerTracker* animal feeding behaviour and food intake across genotypes and light conditions (i.e., LD vs DD, Figure 7E–H), combining both male and female data (which is disaggregated in ED - 11 ^23^ and provided as individual traces in ED - 11 ^23^). Rates of animal-device interactions and feeding are greater during the dark phase than in the light phase as expected for nocturnal animals (i.e., comparing Figure 7A–B, F(1,8)=19.47, p=0.0022 for animal-device interactions; and Figure 7C–D, F(1,8)=63.51, p<0.0001 for feeding). This is reflected as a general increase in rates of activity and feeding in DD in Figure 7E and Figure 7F, respectively. In LD, while overall animal-device interactions across 24h appears the same in control and cryptochrome-deficient mice ( Figure 7I, t(10)=0.002571, p=0.9980), cryptochrome-deficient animals show increased activity during the light-phase ( Figure 7A, t(10)=3.174, p=0.0099) with a non-significant trend towards decreased activity during the dark-phase compared to controls ( Figure 7B, t(10)=1.069, p=0.3100). In contrast, LD feeding decreases in cryptochrome-deficient mice across a 24h period compared to controls ( Figure 7K, t(10)=3.466, p=0.0061). This is reflected by a decreased feeding in cryptochrome-deficient mice during the dark-phase ( Figure 7D, t(10)=3.446, p=0.0063) and no difference during the light phase ( Figure 7C, t(10)=0.6044, p=0.5590). 24h overall rates of animal-device interactions do not differ between LD and DD (i.e., comparing Figure 7I–J, t(10)=0.03827, p=0.8498) with no effect of genotype in DD (i.e., Figure 7J, t(10)=0.8282, p=0.4269). However, decreased LD feeding in cryptochrome-deficient mice is not reflected in DD (i.e., a significant difference between trends in Figure 7K–L, t(10)=10.65, p=0.0115) with no difference by genotype ( Figure 7L, t(10)=0.7710, p=0.4585).

**Figure 8.**
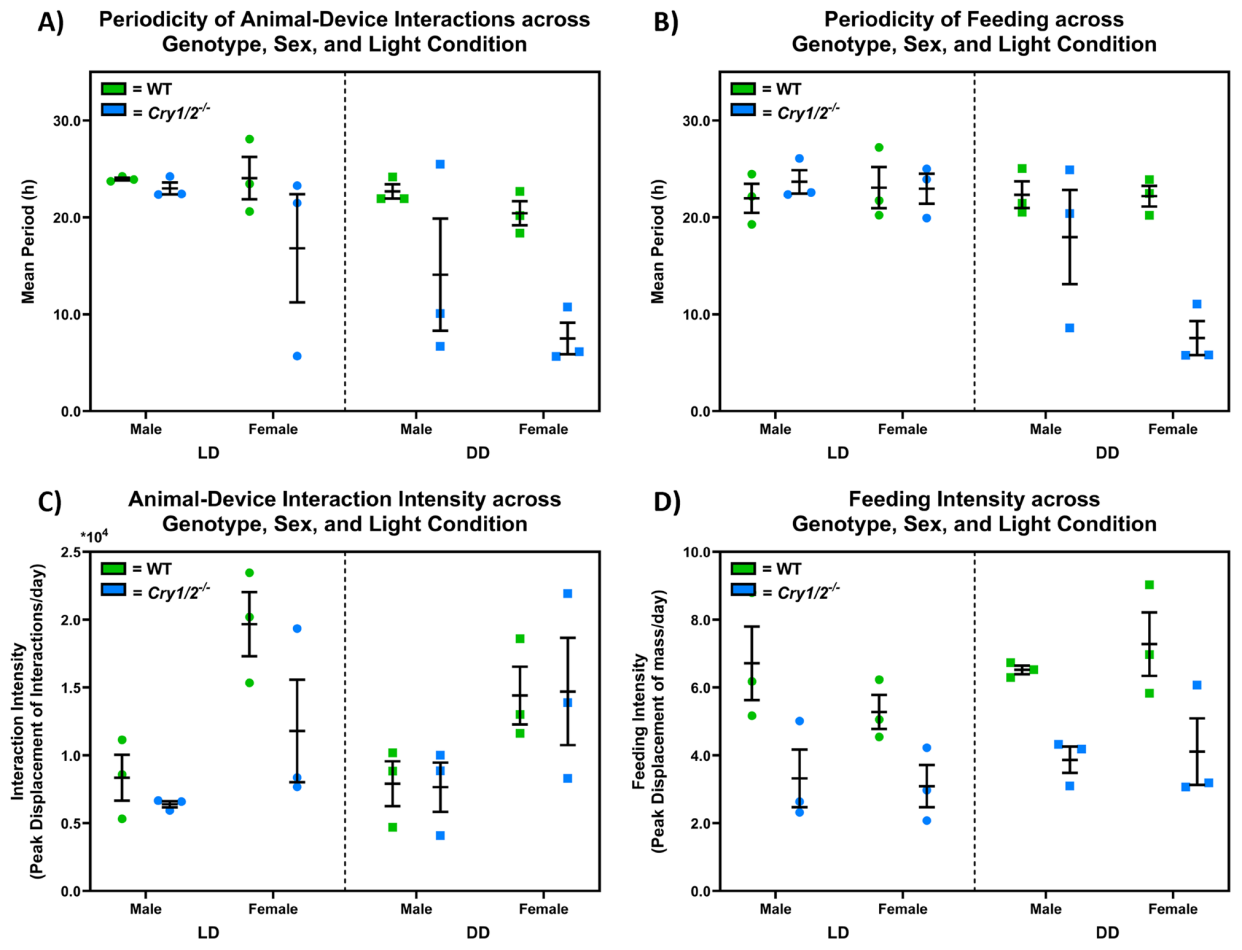
Animal feeding behaviour periodicity and intensity differs by genotype, sex, and home-cage lighting conditions. **A**) The period of animal-device interactions is shorter in cryptochrome-deficient mice ( *Cry1 ^-/-^,Cry2 ^-/-^*) compared to control mice (C57BL/6J) (F(1, 8)=11.51, p=0.0095). This decrease is more pronounced in cryptochrome-deficient mice when kept in constant darkness (DD) compared to in 12:12h light:dark conditions (LD) (F(1,8)=7.344, p=0.0267). There were no statistically significant sex differences (F(1,8)=3.061, p=0.1183). **B**) Similarly, feeding periodicity decreases for cryptochrome-deficient mice in DD in both male and female mice (i.e., a significant overall effect of light condition and genotype, including their interaction, but not sex though the trend appears more prominent in females (F(1,8)=11.03, p=0.0105; F(1,8)=7.934, p=0.0226; F(1,8)=11.07, p=0.0104; and F(1,8)=2.449, p=0.1562 respectively). **C**) Females show more intense animal-device interactions than males (F(1, 8)=21.82, p=0.0016). This is observed in both control and cryptochrome-deficient mice under LD and DD, with no significant effect of genotype or light condition (F(1,8)=1.710, p=0.2273 and F(1,8)=0.05525, p=0.8201 respectively). **D**) Cryptochrome-deficient mice demonstrate decreased feeding intensities compared to controls (F(1,8)=39.07, p=0.0002), which occurs in both males and female (i.e., no sex differences, F(1,8)=0.07726, p=0.7881) under LD and DD (i.e., no significant effect due to light condition, F(1,8)=1.980, p=0.1971).

Figure 8 compares the behavioural periodicity and intensity of *SnackerTracker* feeding and animal-device interactions across genotype, sex, and light conditions. The periodicity of animal-device interactions decreases for cryptochrome-deficient mice in DD ( $\bar{x}$ =24.00h in LD vs $\bar{x}$ =21.55h in DD for controls, yet $\bar{x}$ =19.90h in LD vs $\bar{x}$ =10.80h in DD for cryptochrome-deficient mice, considering both males and females), with a significant overall effect of light condition (F(1,8)=7.344, p=0.0267) and genotype (F(1, 8)=11.51, p=0.0095) but not sex (F(1,8)=3.061, p=0.1183) as shown in Figure 8A. Similarly, Figure 8B suggests that feeding periodicity decreases for cryptochrome-deficient mice in DD in both male and female mice ( $\bar{x}$ =22.52h in LD vs =22.27h in DD for controls, yet $\bar{x}$ =23.31h in LD vs $\bar{x}$ =12.76h in DD for cryptochrome-deficient mice, considering both males and females), with a significant overall effect of light condition (F(1,8)=11.03, p=0.0105), genotype (F(1,8)=7.934, p=0.0226), the light condition × genotype interaction (F(1,8)=11.07, p=0.0104), but not sex (F(1,8)=2.449, p=0.1562). Females show more intense animal-device interactions than males ( $\bar{x}$ =15147 interactions/d for females vs =7578 interactions/d for males, F(1, 8)=21.82, p=0.0016) ( Figure 8C), which is observed in both control and cryptochrome-deficient mice under LD and DD (i.e., no significant effect of genotype or light condition: F(1,8)=1.710, p=0.2273 and F(1,8)=0.05525, p=0.8201 respectively). Figure 8D shows decreased feeding intensity in cryptochrome-deficient mice compared to controls ( $\bar{x}$ =6.45g/d for control mice vs $\bar{x}$ =3.60g/d for cryptochrome-deficient animals, F(1,8)=39.07, p=0.0002), which occurs in both male and female mice (i.e., no effect due to sex, F(1,8)=0.07726, p=0.7881) under LD and DD (i.e., no effect due to light condition, F(1,8)=1.980, p=0.1971).

## Discussion

The *SnackerTracker* is a sensitive tool that continuously measures mouse food intake, feeding behaviour and ambient cage lighting. This provides researchers with insight into both the amount and pattern of food consumption in mice and is suitable for other laboratory rodents (e.g., hamsters). It is novel in its open-source design and hardware/software provision: both were created such that interaction with source files is not required for those with little CAD/programming experience, yet others wanting full command have access to underlying scripts. This provides researchers with a platform wherein both the chassis and processing pipeline can be modified to suit individual experimental needs. For example, the food holder could be re-designed to present food horizontally for compatibility with simultaneous EEG recordings, and additional peripheral inputs such as thermal cameras could be easily incorporated (see ED - 1) ^23, 35^. Both operational and processing software is adaptable to facilitate a range of data collection and filtering according to user-specifications. Validation tests provide good evidence for *SnackerTracker* precision over time in both benchtop and *in vivo* settings. This is supported by accurate measures of light exposure and standard masses both in a controlled laboratory environment and *in vivo* when compared to manual data ( Figure 5). Feeding and activity traces exhibit good correlation to home cage activity recordings ( Figure 6). By testing the *SnackerTracker* using control and cryptochrome-deficient mice with known differences in activity, we demonstrate its ability to track and reveal biologically relevant behaviours related to feeding.

While solutions were found to some challenges encountered during *SnackerTracker* development, others revealed inherent limitations related to data collection, practical application, and both hardware and software device design. Notable considerations related to data collection include IoT monitoring, LDR quality, and measurement drift. Despite successful IoT monitoring during benchtop testing ( Figure 5E and ED - 6 ^23^), the IoT was incompatible with university networks and therefore was not used for *in vivo* recordings (institutional restrictions commonly prohibit IoT device networking due to security concerns). A lack of IoT connection prevents remote monitoring yet does not preclude local SD data storage. *SnackerTracker* LDR measurements are provided in arbitrary units and, whilst these can provide lights on/off information, they cannot discern absolute intensities or wavelengths. Although data drift was not observed during testing, this should not be assumed and if present would require additional data filters. Drift may onset with device wear, longer recording times, and under varied experimental conditions (e.g., temperature and humidity fluctuation) ^31^ and should therefore be confirmed periodically (recommended monthly if in frequent use, and before any set of recordings if latent for more than one month). More sophisticated signal processing algorithms should be considered to assess more nuanced waveform features, which may reveal insight into distinct circadian phenotypes ^36^ that currently cannot be detected by the Curve Fitting Toolbox’s Sum of Sine regression model.

Additional practical limitations including social housing, crumbling, and prolonged non-feeding interactions with the food holder which affect the accuracy of *SnackerTracker* data. The current design is solely suited to single-animal housing as one cannot otherwise discriminate when and how much pellet removal could be attributed to multiple animals. Systematic error is likely also present due to crumbling through the removal of pellet portions which are not being imminently eaten (e.g., for hoarding and later consumption, which then cannot be tracked). Though this was to proportionally little effect at $\bar{x}$ =0.219±0.016g/d (≈5%), both crumbling and active removal, chewing, and discharge onto the cage floor may erroneously inflate measured food consumption especially if the expected difference between conditions is ≤5%. Crumbling is a well-established phenomenon which advanced caging systems have been designed to circumvent (e.g., a cage-floor size-exclusionary sieve) ^12^. This was not addressed with *SnackerTracker*, yet future cage setups could be adapted to reduced effects. Additionally, animals may sit, defecate, or place bedding and sawdust on the food holder, which can cause non-negligible artificial decreases in the recorded mass of food consumed. The 'chip filter' was therefore created in the analysis scripts where, during filtering step four (ED - 8–9 ^23^), sharp spikes followed by steep dips can be manually identified, attenuated, and documented in output metadata (ED - 10 ^23^). 

Challenges which emerged related to device design include recording duration, wear over time, and the manner of food presentation. Wireless *SnackerTracker* operation is required for IVC compatibility yet is limited by a maximum battery life of approximately 2.5 days without IoT/Wi-Fi connectivity (which would otherwise dramatically increase current draw). In anticipating longer recordings in open-top cages, the case cover was adapted such that a grommet aligns with the Arduino MKR 1010 USB port and USB power can be supplied. This was piloted in open-top cages wherein continuous week-long recordings were collected without complication. Protocols for both wired and wireless data collection are distinguished accordingly in protocols.io ^28^. Furthermore, device longevity is a concern as mice naturally chew and scratch. Though damage was minimal after six months of use, printing or casting the chassis using more durable materials (e.g., aluminium) could preserve *SnackerTracker* integrity, though with an increased cost. The prospect of urine damaging electronics was concerning, however was circumvented by the beveled design of the case cover insert (see ED - 2-I ^23^). When testing USB power supply, one mouse chewed the wire at the grommet interface. Simply wrapping electrical tape around this region prevented further chewing, however this may not suffice if mice chew more aggressively. As with any recording device in the home cage, researchers should ensure that animal access to any wires or sensitive components is limited (which may depend on the cage setup), and that the device is regularly inspected for damage.

Characterising feeding behaviour and food intake in mice with disrupted circadian rhythms (e.g., cryptochrome-deficient) can complement metabolic interpretations in a range of disease-relevant applications. Differences in LD vs DD activity has been previously studied in cryptochrome-deficient animals ^37, 38^ and other models having arrhythmic or disrupted circadian rhythms, such as Period 1 and 2 double knockout mice ( *Per1 ^-/-^,Per2 ^-/-^*) ^9, 39, 40^, Bmal1 knockout mice ( *Bmal1 ^-/-^*) ^8, 10, 41^, and CLOCK mutant mice ^42– 44^. In LD, cryptochrome-deficient mice eat less and weigh 10–20% less than control animals ^45, 46^, exhibit perturbed lipid metabolism ^47^, and rapidly gain weight when exposed to high fat diets ^46^. This has made the cryptochrome-deficient mouse an important model in assessing the role of circadian disturbance in disorders including obesity and metabolic syndrome ^46, 48, 49^. Rhythmic expression of some hepatic enzymes associated with feeding behaviour persist even in the absence of an intact circadian clock, which in turn influences global clock gene transcription ^11, 39, 50, 51^. This may compensate for selective clock gene deficiency and therefore confound the interpretation of interventions to circadian dysregulation (e.g., altered diets ^46, 52– 54^, drug administration ^55– 57^, or scheduled feeding.

In this study, *SnackerTracker* recordings identify differences between cryptochrome-deficient and control mice in both the amount and time-course of animal feeding behaviour and food intake. While animal-device interactions do not differ across genotypes, arrhythmic mice feed less in the dark-phase during LD. This may be due to reduced food intake in smaller animals as control animals weighed more than cryptochrome-deficient mice. Conversely, it is possible that animals which eat less will be lighter. However, this difference in feeding between genotypes was not observed in DD (p=0.4585), where WT food intake decreased to approximately the same amount as that which was consumed by cryptochrome-deficient mice (no change in the amount of food intake was observed for cryptochrome-deficient mice in LD or DD). Control animals maintained similar daily rhythms in DD as in LD, whereas cryptochrome-deficient animals exhibit irregular bouts of activity in DD. This aligns with previous literature and demonstrates that the *SnackerTracker* can detect behavioural changes in activity and feeding.

As expected, control animals show similar activity and feeding patterns in both LD and DD, while cryptochrome-deficient mice demonstrate rhythmic behaviours in LD which are lost in DD. Unexpectedly, cryptochrome-deficient mice appear to show ultradian rhythms (<24h) in activity and feeding rates in DD (τ≈10.80h and τ≈12.76h, respectively). A possible underlying mechanism might relate to dopaminergic ultradian oscillations (DUOs) promoting alertness, which has been shown to prominently influence behavioural patterns in voles ^58^. Ultradian rhythms are independent of the canonical circadian clock as DUOs persist following SCN lesion ^59^, with dopamine levels increasing and decreasing in tandem with activity levels ^38^. Therefore, DUOs are a candidate driver of daily rhythms in cryptochrome-deficient mice in LD or DD, wherein removing more prominent circadian-like effects of external light cues in DD conceivably unmasks underlying ultradian oscillations.

When examining individual datasets (ED - 11 ^23^), these DD feeding bouts can be observed in all cryptochrome-deficient mice, yet appear shorter and more prominent in females (periods of ≈7.54h with an amplitude of $\bar{x}$ =15147 interactions/d for females, vs ≈17.97h with an amplitude of vs $\bar{x}$ =7578 interactions/d for males). These sex differences are not obvious in the combined timeline ( Figure 7). More intense female interactions compared to males has been previously observed in longitudinal actogram experiments ^60^, and the observed periodicity of ≈17.97h in male cryptochrome-deficient mice closely resembles the pattern of running-wheel activity observed by Putker *et al.* They report that male cryptochrome-deficient mice exhibit an average period of ≈17h, with greater variance than WT controls ^38^, however, this previous study only considered effects in male mice ^38^. Differences observed between male and female mice in this study could be simply attributed to high random variability, true sex-differences, or both.

Contrary to historical arguments to only include male mice in experimental studies due to estrous cycle-induced variability in females (approximately every 4–5 days), male mice exhibit *more* variance in temperature and activity data than females *within* 24h days ^61^. This aligns with the difference in variability observed in the present study (i.e., fitted curves for male data exhibit greater sum-of-squares differences and coefficients of determination compared to fitted curves for female data—see underlying data and ED - 12 ^23^). Furthermore, recent studies show that female mice exhibit more pronounced and frequent daily temperature oscillations during the estrus phase—particularly in the dark phase—suggesting that estrogen enhances oscillatory temperature dynamics ^62^. When estrogen levels peak, female mice exhibit increased locomotor activity and body temperature fluctuations ^62^, which may be associated with elevated metabolic demands during this phase. Under otherwise normal conditions, murine temperature, feeding, and activity timelines closely correlate when timelines are aligned and compared ^36, 63– 65^. Therefore, feeding and activity data exhibiting shorter periods and more prominent amplitudes observed in this study might have occurred due to higher levels of circulating estrogen in female mice. This could particularly explain observed sex-differences in DD as a combination of unmasking underlying rhythms in the absence of external light cues and increased estrogen-related temperature oscillations in darkness ^62^. This does not preclude—but rather compliments—DUO conjectures; estrogen has been shown to increase dopamine levels, the number of dopamine receptors, and the affinity of dopamine transporters in the brain ^66^. Therefore, these sex-differences in observed activity bouts could be driven by a range of physiological factors related to DUOs, body temperature, appetite, and metabolic needs, yet is more likely attributable to a combination of these. Altogether, this provides evidence and possible explanations for sex-dependent ultradian rhythms in cryptochrome-deficient animals.

Any experimenter must also consider whether *SnackerTracker* food presentation (i.e., compared to the cage-floor or food-hopper) influences feeding behaviour or food intake in genetically modified or ill animals. The final design presents food in a manner which resembles normal cages with food hoppers; any mouse line that struggles to access the food hopper in a normal cage should similarly struggle to access food from the *SnackerTracker*. Supplementing the cage floor with pellets or mash would render *SnackerTracker* recordings invalid, however *SnackerTracker* feeding would then resemble restricted feeding if no food supplements were provided (which should never occur unless built into experimental plans and ethically approved). Similar considerations should be made when working with animals with putative anxiety or feeding-related phenotypes ^67^, as neophobic animals may eat less when presented with a novel food source. As such, alternative physiological and behavioural interpretations of all *SnackerTracker* outputs should always be considered. Animals should be monitored closely during recordings, including body weight and general welfare documentation, especially when first exposed to this new feeding system.

In future applications, the *SnackerTracker* could be used in a range of home-cage setups, with other animal species (we have successfully tested this in hamsters), and in combination with other monitoring systems according to study-specific requirements. For example, feeding and activity data could be correlated to body temperatures over time to test the sex-dependency of feeding activity oscillations as a function of estrous cycles and temperature profiles. This could be achieved by obtaining thermal data via implanted devices or non-invasive imaging ^35^, and activity via passive infrared ^15^ or video recordings. These parallel datasets could validate *SnackerTracker* recordings and provide additional insights into behaviours influencing data interpretation. Similarly, the *SnackerTracker* could easily be combined with indirect calorimetry to relate metabolic parameters to feeding behaviour. The *SnackerTracker* was designed for expansion and customisation, for instance to become compatible with studies involving head-mounted electroencephalography/electromyography leads, optogenetic implants, or multielectrode probes. The food holder could be re-designed such that pellets extend horizontally to prevent overhead wire-tangling. Demand will drive future directions, which are wide in scope due to the *SnackerTracker*’s adaptable design, low cost, and open-access provision ^26^.

Multiple refinements to animal experiments may be achieved using the *SnackerTracker*. Documenting feeding patterns is beneficial when monitoring animal welfare, wherein real-time *SnackerTracker* IoT-based monitoring would be particularly beneficial. For example, collecting data may be useful after surgical procedures to ensure normal recovery as reduced appetite will typically accompany sickness. *SnackerTracker* measurements of food intake could be incorporated into welfare assessments, wherein abnormal feeding patterns or increased/decreased food intake could serve as an early indication of disease or experimental complication. Future studies could use the *SnackerTracker* to evaluate food preferences by providing two or more devices in one cage, or by upgrading the design to possess two load cell-food hopper assemblies (with different food provided within each) for simultaneous and self-contained measurement. Data could furthermore be used as an experimental outcome when comparing control mice to those modelling a range of human diseases, for example decreased feeding following digestive disturbance using the dextran sodium sulfate colitis model ^68^, or increased feeding using models of Prader-Willi Syndrome ^69^.

The *SnackerTracker* could furthermore improve the accuracy of drug delivery via diet. Knowing how much drug-laden food an animal has eaten at any point in time would enable researchers to precisely calculate drug dosage for experimental assessment. In animal research, clozapine n-oxide (CNO) and tamoxifen are drugs which are being increasingly used to elicit neuronal responses or trigger gene expression, respectively. CNO activates genetically engineered inhibitory or excitatory designer receptors exclusively activated by designer drugs (DREADDs) expressed in specific tissues or neuron populations ^70^, while tamoxifen triggers gene expression in modified mouse lines ^71^. CNO and tamoxifen are typically injected into mice, yet companies (e.g., Scientific Animal Food & Engineering, Inotiv, Research Diets, Inc. etc.) have begun producing food pellets which provide such drugs in pellet-form (tamoxifen is readily available, and CNO efforts are ongoing). Administering tamoxifen or CNO via diet would be an immense welfare improvement, yet dosage amounts would be difficult to ascertain if food is provided *ad libetum*. Therefore, the *SnackerTracker* would be a valuable means of determining the mass of CNO/tamoxifen-laced food pellets consumed at any time; by knowing the concentration of each drug in the pellets, the amount of CNO or tamoxifen ingested could be calculated. This would be critical whether using drugs for biological research applications or pharmacological development.

*SnackerTracker* recordings would be highly relevant in testing pharmacological candidates related to a wide range of therapies, including but not limited to diabetes, digestive disruption ^11, 68, 72– 75^, weight loss, eating disorders ^69^, metabolic insufficiency, and sleep disturbance. If a researcher is using animal models to test the efficacy of a drug candidate, it is critical to characterise if and how the drug influences feeding whether as an intended or unintended consequence. Importantly, food-intake may not precisely correspond with commonly used measures including actograms of food-anticipatory behaviour and time-courses of respiratory exchange ratios, therefore a dedicated means of measuring feeding is warranted ^44, 76^.

Altogether, we demonstrate the compatibility of the *SnackerTracker* in normal IVC cages as well as its validity compared to established methods of home cage activity recordings (DVCs). We reveal new insights into the feeding behaviour of cryptochrome-deficient mice, including evidence for ultradian feeding patterns, which has important implications for the interpretation of previous and future studies investigating behavioural rhythms in this model. We emphasise the importance of measuring food intake in animal studies to gain welfare and experimental insights, as well as *SnackerTracker* suitability and adaptability across a range of future applications.

## Conclusions

The *SnackerTracker* is a novel animal home-cage monitoring system which continuously records food-intake, feeding behaviour, and ambient light conditions. This paper describes its development, validation, and *in vivo* application. Measurement accuracy is confirmed using both manual and established home cage activity monitoring methods. All material information, 3D-printable files, electronics schematics, construction details, and operational/analysis software are provided on open-access platforms such that researchers can download, customise, and build the *SnackerTracker* according to experiment-specific requirements. Here we show that the *SnackerTracker* provides insights into the behaviour of cryptochrome-deficient animals, identifying light suppression of food intake in rhythmic but not arrhythmic mice and detecting ultradian rhythms in the control of feeding behaviour. The ability of *SnackerTracker* to reveal subtle biological differences, alongside its low-cost and ease-of-operation, demonstrates its usefulness as a methodological refinement to animal home-cage monitoring.

## Ethics

All experiments comply with the UK Animals (Scientific Procedures) Act (1986), Oxford’s Policy on the Use of Animals in Scientific Research, and the ARRIVE guidelines 2.0 ^77^. Procedures were approved by Oxford’s Animal Welfare and Ethical Review Board and performed under valid personal and project licenses (PIL-I84222165, approved December 31, 2021; PPL-PP0546018, approved June 21, 2022; PPL-P828B64BC-PP6154904, approved June 27, 2022; and PPL-PP0911346, approved December 21, 2020). All efforts were made to prevent and minimise animal suffering according to the principles of the 3Rs (Replacement, Reduction, and Refinement). No animals were bred specifically for these experiments (i.e., were surplus and/or following exclusive use in non-aversive behavioural monitoring studies), and the absolute minimum number required was used according to *a priori* power calculations (see Statistics). During all experiments mentioned in this manuscript, at no point was an animal subjected to aversive tests, handling, or cage conditions (aside from single housing, wherein only animals which were previously singly housed were used). Animals underwent daily welfare checks to ensure good health, cage cleanliness, and food/water supply.

## Data Availability

Figshare: *Underlying data - The SnackerTracker: A novel home-cage monitoring device for measuring food-intake and food-seeking behaviour in mice*, https://doi.org/10.6084/m9.figshare.28191125 ^78^. This project contains the following underlying data: Data file 1. *WellcomeOpenResearch - SnackerTracker - CRY Experiment Details.* Referencing corresponding [columns] in this spreadsheet, this data details: Cohort ID [A]; light-dark conditions [B,K]; recording start and end times [C-D]; assigned study numbers and IDs per animal [E,H,I]; body weights before and after recordings; age [F-G]; age [J]; genotype [L]; sex [M]; analysis filter notes [N]; food consumption summary data as analysed from SnackerTracker recordings [O-S]; food-seeking behaviour summary data as analysed from SnackerTracker recordings [T-X]; food consumption as measured manually without the SnackerTracker in the food hopper [Y,AE]; SnackerTracker battery performance notes [Z]; whether independent manual mass measurements were obtained before or after SnackerTracker recordings [AA]; food consumption as measured manually during SnackerTracker recordings [AB] ( Figure 5Fi); and food consumption as measured manually without the SnackerTracker in the home-cage ( Figure 5Fii) [AD]. Rows 27–55 contain summary data. Data file 2. *WellcomeOpenResearch - SnackerTracker - CRY Data Collection Details*. Experiment logbook, with major features collated in Data file 1. Data file 3. WellcomeOpenResearch - SnackerTracker Calibration - Automated - Range. Raw data obtained when testing automated *SnackerTracker* calibration validity ( Figure 5A). Data file 4. *WellcomeOpenResearch - SnackerTracker-Drift-Test2*. Raw data collected during the drift test ( Figure 5B). Data file 5. *WellcomeOpenResearch - SnackerTracker-Drift-Test2-Processed.* Processed data from the drift test ( Figure 5B). Data file 6. *WellcomeOpenResearch - SnackerTracker-Step-Test*. Raw data collected during the step test ( Figure 5C). Data file 7. *WellcomeOpenResearch - SnackerTracker-Step-Test-Processed*. Processed data from the step test ( Figure 5C). Data file 8. WellcomeOpenResearch - SnackerTracker-LDR-Test. Raw data collected during the LDR test ( Figure 5D). Data file 9. *WellcomeOpenResearch - SKRTKR-Combined-Test1.* Raw data collected during the combined validation test (#5, Figure 5E). Data file 10. *WellcomeOpenResearch - SKRTKR-Combined-Test1-Processed.* Processed data from the combined validation test (#5, Figure 5E). Data file 11. *WellcomeOpenResearch - SnackerTracker - Crumbling Tests.* Raw data collected in testing the amount of crumbling during *SnackerTracker* recordings. Data files 12–35. *SKRTKR_CRY_[LD||DD]_[WT||KO]_[M||F]_C[1–11]_00[1–6].* Raw data collected during *in vivo* experiments, with items in brackets denoting (from left to right) light-dark condition, genotype, sex, cohort, and animal study ID corresponding to each file ( Figure 6– Figure 8 and ED 11–12). Data file 36. *WellcomeOpenResearch - SnackerTracker-DVC-Data-All.* Raw outputs from DVC recordings (containing data from all cohorts and animals) ( Figure 6). Data files 37–84. *SKRTKR_CRY_[LD||DD]_[WT||KO]_[M||F]_C[1–11]_00[1–6].TXT-[Process||Processed]-[Compressed||Parameters].* Outputs of *SnackerTracker* data processing, for each of 24 files containing one metadata/summary output file (appended ‘Parameters’) and one compressed data file (appended ‘Compressed’) ( Figure 6– Figure 8 and ED 11–12). Data file 85. *WellcomeOpenResearch - SnackerTracker-Mean-Period-Interactions*. Consolidated sin-fitting outputs detailing the average period of the best-fit curve for food-seeking behaviour per experimental condition (i.e., period of feeding behaviour) ( Figure 8A). Data file 86. *WellcomeOpenResearch - SnackerTracker-Mean-Period-Feeding.* Consolidated sin-fitting outputs detailing the average period of the best-fit curve for food intake per experimental condition (i.e., period of feeding) ( Figure 8B). Data file 87. *WellcomeOpenResearch - SnackerTracker-Mean-Intensity-Interactions*. Consolidated sin-fitting outputs detailing the average amplitude of the best-fit curve for food-seeking behaviour per experimental condition (i.e., intensity of feeding behaviour) ( Figure 8C). Data file 88. *WellcomeOpenResearch - SnackerTracker-Mean-Intensity-Feeding*. Consolidated sin-fitting outputs detailing the average amplitude of the best-fit curve for food intake per experimental condition (i.e., intensity of feeding) ( Figure 8D). All data are available under the terms of the Creative Commons Zero “No rights reserved” data waiver (CC0 1.0 Public domain dedication). Figshare: *Extended data - The SnackerTracker: A novel home-cage monitoring device for measuring food-intake and food-seeking behaviour in mice*, https://doi.org/10.6084/m9.figshare.28407689.v1 ^23^. This project contains the following files: ED - 1 - *SnackerTracker* Design Criteria and Constraints. Lists design criteria and constraints for *SnackerTracker* development. Requirements are classified as ‘primary’ (green) = mandatory for device function; ‘secondary’ (blue) = should be satisfied to meet the needs of the present study; or ‘tertiary’ (yellow) = an advantage to have, but not necessary, and worth considering for future applications. ED - 2 - *SnackerTracker* Chassis Schematics for Parts I-IV. Provides technical drawings for each chassis component including the I - body, II - case cover, III - mount plate, and IV - food holder. ED - 3 - *SnackerTracker* 3D Printing Specifications. Lists custom settings used within Ultimaker Cura when 3D printing the *SnackerTracker* chassis (optimised through iterative testing). Automatically assigned values were maintained for any additional parameters not listed here. Parameters are grouped by print features influencing chassis body quality and composition (green); overhang and build plate supports which are later removed (blue); and the mesh, which is an immaterial computational construct (yellow). ED - 4 - *SnackerTracker* Bill of Materials Extended Version. The Bill of Materials (BOM) lists system components, mounting/accessory elements, and tools needed to build one device. This sheet also provides information detailing the exact or approximate quantity of each product needed to build one device, as well as links to supplier websites as available at the time of publication. Some components listed under ‘Construction and Testing’ and ‘Accessories’ are not considered in price calculations as these can be re-used across other devices/projects. The material cost for one device at the time of publication totaled just under £120, including VAT (less if parts are ordered in bulk or with VAT deduction). ED - 5 - *SnackerTracker* Electronics Circuit Diagram. Illustrates the schematic circuit diagram corresponding to *SnackerTracker* electronics. ED - 6 - *SnackerTracker* IoT User Interface. A) Shows the IoT ‘thing’ configuration, where users may customise device control and ensure Wi-Fi connectivity. The user can configure multiple devices and operational settings according to experimental requirements. The user can easily navigate between this and the B) dashboard for remote data-monitoring. These can be visualised on any connected device, including via the C) mobile app and D) corresponding interface. ED - 7 - *SnackerTracker* Software Variables. Lists and describes *descriptive* variables (green), *processing* variables (blue), and *control* variables (yellow) used during *SnackerTracker* data analyses. Descriptive variables register experiment information for recordkeeping and do not influence underlying data. Processing variables actively filter datasets; effects of increasing or decreasing these are described, and sample values are provided. Control variables act as checkpoints throughout stages of data-filtering. The data processing step (1–9) at which each variable is initialised is provided for reference. ED - 8 - *SnackerTracker* Data Processing Steps. Lists and briefly describes the nine main steps involved with *SnackerTracker* data processing. Corresponding figures are provided in ED - 9. ED - 9 - *SnackerTracker* Data Processing Steps Visualised. Provides figure references for the nine main steps involved with *SnackerTracker* data processing. These include: i) raw data and switch assessment; ii) crop specifications; iii) mass filtering; iv) plateau filter application; v) interactions assessment and frequency analysis; vi) LDR filtering; vii) output summary, viii) cropping/alignment, and ix) data compression. ED - 10 - Sample *SnackerTracker* Outputs. Depicts representative *SnackerTracker* output files following data processing, where green = summary information as obtained from the entire recording sample (from the spreadsheet appended ‘Process-Parameters’); blue = processing specifications (also from spreadsheets appended ‘Process-Parameters’); and yellow = sample timeline outputs, transposed and truncated here for brevity (from spreadsheets appended ‘Processed-Compressed’). Processed data which has *not* been compressed is simply appended ‘Processed’; however, these spreadsheets are not compatible with some graphing programmes (e.g., GraphPad Prism, v10.2, RRID:SCR_002798) if they contain an excessive number of data points. ‘Processed-Compressed’ files are therefore useful. ED - 11 - Control and Cryptochrome-Deficient Behavioural Data Disaggregated by Sex. This illustrates the same data as shown in Figure 7 of the corresponding manuscript, which shows that control (wild-type, C57BL/6J) and cryptochrome-deficient ( *Cry1 ^-/-^,Cry2 ^-/-^*) animal activity and food intake differs across LD and DD conditions, but disaggregated by sex. Females appear to be more active than males. E) In DD, more prominent activity bouts can be observed for female cryptochrome-deficient mice as opposed to males or control mice of both sexes. No notable sex-differences are observed for food consumption. Timeline plots and summary data are presented as $\bar{x}\pm \sigma_{M}.$ ED - 12 - Control and Cryptochrome-Deficient Behavioural Data as Individual Traces. Provides all raw A) animal-device interaction and B) feeding traces for control (wild-type, C57BL/6J) and cryptochrome-deficient ( *Cry1 ^-/-^,Cry2 ^-/-^*) animals under both LD and DD conditions, underlying ED - 11 and Figure 7 of the corresponding manuscript. All files are available under the terms of the Creative Commons Zero “No rights reserved” data waiver (CC0 1.0 Public domain dedication).

## Software availability

Source code is available from: https://github.com/marhmue/SnackerTracker-Code---Core

Archived software is available from: https://doi.org/10.5281/zenodo.14632999 ^29^

License: All software is available under the terms of the GNU General Public License v3.0.

### 3D models

3D models (and the corresponding build) have been uploaded to the NIH 3D Print Exchange: *SnackerTracker* model ID #3DPX-021724 ( https://doi.org/10.60705/3dpx/21724.1) ^27^ and build ( https://3d.nih.gov/builds/657). All materials are available under the Creative Commons Attribution-NonCommercial License (CC BY NC).

### Overview video and user resources

A summary video with subtitles as well as instructional videos corresponding to the methods described in this manuscript are available via Figshare: *Video Resources - SnackerTracker Overview and User Guidance* at https://doi.org/10.6084/m9.figshare.28189793 ^24^. Contents are available under the Creative Commons Attribution 4.0 International License (CC BY 4.0).

Step-by-step instructions are also available on protocols.io: *SnackerTracker* at https://dx.doi.org/10.17504/protocols.io.rm7vzke8rvx1/v1 ^28^. Contents are available under the Creative Commons Attribution 4.0 International License (CC BY 4.0).

The SnackerTracker has also been uploaded as an Arduino Project on the Arduino Project Hub at https://projecthub.arduino.cc/marissamueller/snackertracker-d86bf3, and available under the Creative Commons Attribution-NonCommercial License (CC BY NC).

### Reporting guidelines

Adherence to reporting standards is documented in the ARRIVE guidelines 2.0 checklist ^77^ deposited to Figshare: *Reporting guidelines - The SnackerTracker: A novel home-cage monitoring device for measuring food-intake and food-seeking behaviour in mice* at https://doi.org/10.6084/m9.figshare.28407746 ^79^. Data are available under the Creative Commons Zero “No rights reserved” data waiver (CC0 1.0 Public domain dedication).
