# Supplementary material for: The SnackerTracker: A novel home-cage monitoring device for measuring food-intake and food-seeking behaviour in mice
Source: Wellcome Open Res. 2025 Jul 23;10:172. Originally published 2025 Apr 4. [Version 2] doi: 10.12688/wellcomeopenres.23850.2 (PMC12375189; doi:10.12688/wellcomeopenres.23850.2)
Supplement: Peer review history

# Reviewer report: Reviewer response for version 2

**DOI:** 10.21956/wellcomeopenres.27194.r128551

**Published:** 2025-09-02

**Authors:** Raoni Conceição dos-Santos

**Affiliations:** 1 University of South Carolina School of Medicine, Columbi, South Carolina, USA

In this paper the authors discuss the development and validation of the Snackertracker, a low-cost automated system to measure food intake, food seeking activity and ambient light conditions. The equipment is designed so that it can be added to the normal pre-existing cages, keeping the cost much lower than other similar equipment. The authors provide extensive and detailed instructions and open-source blueprints and software to allow for replication of the device by interested parties, granted if they possess the necessary engineering knowledge to build it. Furthermore, the authors thoroughly validated the device by comparing the results in the Snackertracker with a conventional equipment and by assessing the changes in food-related behaviors in known models of circadian disturbances.

 The authors have addressed the concerns of previous reviewers. I am interested in the device and would like to implement it in my future studies.

 Minor suggestions:

 - Perhaps stating "Cry1-/-, Cry2-/-" is too technical for the plain language summary. This could be removed in following versions.

 - In figure 1, it would be helpful to add a link to the legend, so that the reader can click and download the video in the same device he is reading the paper. 

 - At page 12, "informed the value to which the band-pass filter (bufferHere) should be initialised (i.e., 2×0.045≈0.1g, see ED - 723)."  What do the authors means by "bufferHere", this looks like a typo.

 - In page 14 the authors state: "with a non-significant

 trend towards decreased activity during the dark-phase compared to controls (Figure 7B, t(10)=1.069, p=0.3100).". I would argue that a p value of 0.3 is not a trend.

Is the rationale for developing the new method (or application) clearly explained?

Yes

Is the description of the method technically sound?

Yes

Are the conclusions about the method and its performance adequately supported by the findings presented in the article?

Yes

If any results are presented, are all the source data underlying the results available to ensure full reproducibility?

Yes

Are sufficient details provided to allow replication of the method development and its use by others?

Yes

Reviewer Expertise:

Rodent behavioral essays, food intake behaviors, sickness behaviors.

I confirm that I have read this submission and believe that I have an appropriate level of expertise to confirm that it is of an acceptable scientific standard.

# Reviewer report: Reviewer response for version 2

**DOI:** 10.21956/wellcomeopenres.27194.r127678

**Published:** 2025-08-23

**Authors:** Michael C Antle

**Affiliations:** 1 University of Calgary, Calgary, Alberta, Canada

Nice work on the revisions, I have no further comments.

Is the rationale for developing the new method (or application) clearly explained?

Yes

Is the description of the method technically sound?

Yes

Are the conclusions about the method and its performance adequately supported by the findings presented in the article?

Yes

If any results are presented, are all the source data underlying the results available to ensure full reproducibility?

Yes

Are sufficient details provided to allow replication of the method development and its use by others?

Yes

Reviewer Expertise:

Rodent circadian rhythms, including feeding behavior

I confirm that I have read this submission and believe that I have an appropriate level of expertise to confirm that it is of an acceptable scientific standard.

# Reviewer report: Reviewer response for version 1

**DOI:** 10.21956/wellcomeopenres.26313.r122983

**Published:** 2025-05-26

**Authors:** Michael C Antle

**Affiliations:** 1 University of Calgary, Calgary, Alberta, Canada

The authors have designed, built, tested and validated a novel feeding tracking system. The system is open source, and is easy and inexpensive to build and can be easily customized for a variety of settings and needs. This is a useful product, and will find application in many settings.  The manuscript is well written and provides enough detail to understand its assembly and operation.

 I only have a couple points for the authors to address:

 1. The main goal of this equipment is to track interaction with the food, and to monitor food "consumption". The key technology that permits these measurements appears to be the inclusion of the load cell. A short paragraph highlighting this component, how it works and how its inclusion in the design allows these various measures to be made would assist the reader in understanding the operation of the Snacker Tracker.

 2. How might the moisture content of the food and the relative humidity of the environment influence measures over time (food increasing in weight as it absorbs moisture in humid environments, or food weighing less as moisture is lost to a dry environment)

 3. A photo of the Snacker Tracker is presented in figures 1+3, and photos of the cages are presented in Figure 2. A photo of both together  (i.e., the snacker tracker interfaced with a cage) would help understand the operation of this equipment.

 4. On page 9-10, the authors discuss events that led to exclusion of data. In cases where this was related to operation of the equipment, a section of the discussion could be dedicated to discussing these events, as others who want to implement this technology might want to anticipate and mitigate similar occurrences.

 5. The first couple sections of the methods section are VERY detailed, pretty much step by step assembly instructions. This might be a little to detailed for the main manuscript, and could be included with all the other online materials. It is more important to discuss what is incorporated and why.

 6. The drift test is a nice inclusion. One source of drift I've often noticed is how the device responded to a bump. Adding a load will cause it to deviate while the load is applied, but how reliable is the device at returning to the pre-load state? A simple test applying a load and then removing it could address such an event might lead to drift.

 7. Bottom of page 8, 4th last line, check the Beta-1 value, it appears to be a repeat from a few lines earlier.

 8. There are two switches included in the design, and they are mentioned in figure 5E. It is not clear to me what these switches are meant to do. Perhaps the text around their function could be enhanced in the relevant locations.

 9. figure 7 C+D show food consumption in the light (C) and dark (D) of an LD cycle. These sum to about 8.5g over both  L+D for the WT mice. In Figure 7D, the mean daily consumption in LD for WT mice is about half this (~4.25g). Since their is about 200mg  food loss due to crumbling, understanding the daily consumption is important if one were to calculate what % the crumbling  loss represents of the full daily amount.

 10. Many circadian researchers are interested in "food restriction" and "Time-restricted feeding". The authors discuss food restriction briefly on page 18, but it is not clear what they mean when they write: "Supplementing the cage floor with pellets or mash would render *SnackerTracker* recordings invalid, however *SnackerTracker* feeding would then resemble restricted feeding if no food supplements were provided" .

Is the rationale for developing the new method (or application) clearly explained?

Yes

Is the description of the method technically sound?

Yes

Are the conclusions about the method and its performance adequately supported by the findings presented in the article?

Yes

If any results are presented, are all the source data underlying the results available to ensure full reproducibility?

Yes

Are sufficient details provided to allow replication of the method development and its use by others?

Yes

Reviewer Expertise:

Rodent circadian rhythms, including feeding behavior

I confirm that I have read this submission and believe that I have an appropriate level of expertise to confirm that it is of an acceptable scientific standard, however I have significant reservations, as outlined above.

# Response

**Published:** 2025-07-12

**Authors:** Marissa Mueller

**Affiliations:** Physiology Anatomy and Genetics, University of Oxford Department of Physiology Anatomy and Genetics, Oxford, England, UK

We would like to thank the reviewers for their thoughtful, constructive, and insightful feedback on our manuscript. We appreciate the time and effort invested in providing these suggestions, which have been extremely useful in improving this present work. Below, please find point-by-point responses to each comment, wherein edits have been made in the manuscript accordingly with tracked changes. We believe these revisions have strengthened the manuscript and address all concerns raised. *The main goal of this equipment is to track interaction with the food, and to monitor food "consumption". The key technology that permits these measurements appears to be the inclusion of the load cell. A short paragraph highlighting this component, how it works and how its inclusion in the design allows these various measures to be made would assist the reader in understanding the operation of the Snacker Tracker.* Certainly. We have added a paragraph at the beginning of the results section, with the subtitle ‘overview’. This describes the most critical module of the *SnackerTracker*’s design as the load cell, how this works as a strain gauge to convert mechanical deformation into electrical signals, and how these signals are recorded as mass values. We then describe how these mass values are used to determine the number of animal-device interactions, the mass of food intake, and the derivatives of both which serve as primary output metrics (i.e., feeding and activity rates, respectively). Thank you for pointing this out; we hope this addition now provides clarification to readers. *How might the moisture content of the food and the relative humidity of the environment influence measures over time (food increasing in weight as it absorbs moisture in humid environments, or food weighing less as moisture is lost to a dry environment)* The moisture content of the food and relative humidity might certainly influence measures over time for the reasons you mention. During this study, all mice were maintained in temperature- and humidity-controlled environments (20–24°C, and 45–65% per UK regulations), which helped minimise the chance of this conceivable artifact occurring. However, as you mention, the practical importance of doing so should be emphasised, and even small differenced within this range could have a notable effect (e.g., 45% vs 65% humidity). Elevated humidity could promote water retention, hinder pellet integrity, and accelerate food deterioration/crumbling. This could directly increase pellet mass or indirectly inflate measured food intake in cases of significant crumbling. For any specific diet used for the first time, potential humidity confounds could be addressed by using an empty cage and weighing food at the start and end of a study (and intervals between if needed) to calibrate humidity effects. We have now described this in the second paragraph of the discussion. *A photo of the Snacker Tracker is presented in figures 1+3, and photos of the cages are presented in Figure 2. A photo of both together (i.e., the snacker tracker interfaced with a cage) would help understand the operation of this equipment.* Certainly. In Figure 2, we have provided two sets of angles, where in B-D one can observe *SnackerTracker *placement within a cage. We have furthermore added two videos and an image to the supplemental file available via Figshare, ‘Video Resources - SnackerTracker Overview and User Guidance’ (https://doi.org/10.6084/m9.figshare.28189793.v3, having updated this link in the manuscript) as follows:

- 2025 - SnackerTrackers Wired in LTCs: Illustrates multiple open-top singly-housed mouse cages within light-tight chambers (LTCs), within which the video '2025 - SnackerTracker Mouse Feeding with Wired Cage Setup' was acquired.
- 2025 - SnackerTracker Mouse Feeding with Wired Cage Setup: This short video depicts an individually-housed mouse eating from a wireless SnackerTracker in a standard open-top cage (as shown in the image '2025 - SnackerTrackers Wired in LTCs').
- 2025 - SnackerTracker Mouse Feeding: This short video depicts an individually-housed mouse eating from a wireless SnackerTracker in a standard open-top cage.

This has been updated accordingly in the section ‘Overview video and user resources’, and is cited in paragraphs 2 and 4 of the discussion. *On page 9-10, the authors discuss events that led to exclusion of data. In cases where this was related to operation of the equipment, a section of the discussion could be dedicated to discussing these events, as others who want to implement this technology might want to anticipate and mitigate similar occurrences.* This is helpful to note, thank you. We have added a paragraph in the discussion (paragraph 4) which addresses each case which led to data exclusion, why it occurred, and the steps taken to fix or minimise its occurrence in future experiments. *The first couple sections of the methods section are VERY detailed, pretty much step by step assembly instructions. This might be a little to detailed for the main manuscript, and could be included with all the other online materials. It is more important to discuss what is incorporated and why.* Thank you for this feedback. Accordingly, we have removed supplier specifications in the ‘Electronics and mounting’ and ‘Software and operation’ sections within ‘Methods’, and simply referenced the complete bill of materials and product details at the beginning of ‘Electronics and mounting’. We have substituted this with additional information regarding why each element is being incorporated, and its function within the device. *The drift test is a nice inclusion. One source of drift I've often noticed is how the device responded to a bump. Adding a load will cause it to deviate while the load is applied, but how reliable is the device at returning to the pre-load state? A simple test applying a load and then removing it could address such an event might lead to drift.* Thank you. We have included a simple dataset to demonstrate this, termed the ‘tap test’, which is described alongside the drift test (now collectively referred to as ‘consistency tests). This includes a brief description in #2 of the ‘Validation tests’ section within ‘Methods’; data overlaid in an updated Figure 5 (and caption); outputs explained in the text under the ‘Testing and validation’ section within ‘Results’; corresponding mention in ‘Discussion’ paragraph 2; and underlying data inclusion as Data Files 89 and 90. The ‘Underlying Data’ link has been updated accordingly. *Bottom of page 8, 4th last line, check the Beta-1 value, it appears to be a repeat from a few lines earlier.* Thank you for catching this. We have updated this to the correct value of β-1 = 0.430 as opposed to β-1 = 0.803. *There are two switches included in the design, and they are mentioned in figure 5E. It is not clear to me what these switches are meant to do. Perhaps the text around their function could be enhanced in the relevant locations.* Thank you for pointing this out. We have clarified this in ‘Software and operation’ within ‘Methods’, noting that the toggle switch information serves no current function. However, these switches were nevertheless included in the present device design with future device improvements in mind. For example, toggle switches could be used as a power button or for event-marking, which could include (but is not limited to) cage checks, device refilling, cage changes, or experimental interventions such as injections. *Figure 7 C+D show food consumption in the light (C) and dark (D) of an LD cycle. These sum to about 8.5g over both L+D for the WT mice. In Figure 7D, the mean daily consumption in LD for WT mice is about half this (~4.25g). Since their is about 200mg  food loss due to crumbling, understanding the daily consumption is important if one were to calculate what % the crumbling  loss represents of the full daily amount.* Certainly—given that the total mass of food consumption differs between animals, the percentage of crumbling loss relative to this total food consumption would change in an inverse manner (i.e., if the amount of crumbling is constant, its effect as a percentage of ‘total food intake’ would be greater if ‘total food intake’ is reduced). As you mention, ~200mg of ~4.25g equates to 4.7% loss, where ~200mg of ~8.5g equates to 2.35% loss. In future experiments, if crumbling is monitored, we have noted in paragraph 3 of the discussion that crumbling should be reported as both an absolute mass and percentage of total food intake. *Many circadian researchers are interested in "food restriction" and "Time-restricted feeding". The authors discuss food restriction briefly on page 18, but it is not clear what they mean when they write: "Supplementing the cage floor with pellets or mash would render SnackerTracker recordings invalid, however SnackerTracker feeding would then resemble restricted feeding if no food supplements were provided".* Thank you for highlighting this. We have re-written these sentences, which are now hopefully clearer as follows: “ If animals struggle to access food, this could unintentionally result in restricted feeding (which should never occur unless experimentally planned and ethically approved). In such an instance, easing access to food by supplementing the cage floor with pellets or mash would render *SnackerTracker* recordings invalid, as the *SnackerTracker* would not be able to measure this alternative food intake. The *SnackerTracker *is not meant to make access to food difficult or restricted in any manner and aims to replicate normal cage feeding for mice. Therefore, if there are husbandry problems with feeding requiring changes to normal food access, using the *SnackerTracker *may be inappropriate.” We have additionally mentioned that the *SnackerTracker *could be beneficial in experiments which involve restricted feeding: “ Conversely, the *SnackerTracker* may be ideal for experimentally planned time-restricted feeding as the device can simply be added to the cage during designated feeding times. The *SnackerTracker* may furthermore help with interpreting effects of food restriction, as it would enable researchers to assess the baseline daily food intake of individual animals prior to food restriction.”

# Reviewer report: Reviewer response for version 1

**DOI:** 10.21956/wellcomeopenres.26313.r122981

**Published:** 2025-05-26

**Authors:** Christopher S Colwell

**Affiliations:** 1 University of California, Los Angeles, California, USA

Mueller and colleagues present the development, validation, and deployment of the SnackerTracker, a cost-effective, open-source home-cage monitoring system designed to continuously measure food intake, food-seeking behavior, and light exposure in singly housed laboratory mice. The device integrates a load cell, ambient light sensor, and Arduino-based data acquisition to enable real-time or recorded tracking of feeding events. The system was validated both *in vivo* using control C57BL/6J mice and circadian-disrupted Cry1/Cry2 double-knockout (CryDKO) mice under 12:12 light-dark and constant darkness conditions. The authors demonstrate that the SnackerTracker accurately captures biologically meaningful variation in feeding behavior and rhythms.

 There are a number of strengths to this work. 

 Provides a low-cost, open-source solution adaptable to standard caging systems.

 Detailed design files, protocols, and code repositories are made openly available, enhancing reproducibility and reuse.

 The authors conduct six benchtop tests (e.g., calibration, drift, light detection, time accuracy) and in vivo comparisons to both manual and commercial activity systems.

 Feeding data show high correlation with other methods, confirming device validity.

 The design supports future expansion (e.g., compatibility with EEG setups, integration with thermal or video tracking).

 As described by the authors, software can be easily modified by users with varying programming experience.

 The manuscript is well-written, highly structured, and transparent about limitations, with a full accounting of sample sizes, exclusions, and statistical power.

 Overall, I am excited to try out this system!

 As a future user, I could see a few limitations.

 The current implementation is only compatible with single housing, limiting its application in studies requiring socially housed animals or high-throughput cohorts.  Would it be possible to incorporate RFID or vision-based individual tracking to support social housing.

 Crumbling and pellet manipulation not associated with consumption can inflate intake measurements, which may limit precision in studies with subtle feeding changes.  Maybe a special hardened food pellet would improve measurements?

 The light-dependent resistor records only relative changes, not absolute irradiance or wavelength, limiting precise circadian photometry applications.  Is it possible to improve standardization of light monitoring, possibly by integrating calibrated lux sensors or spectral sensors.

 Assembly requires technical expertise in soldering, programming, and 3D printing, potentially limiting adoption in labs without engineering support.  Would it be possible to partner with a company to provide a preassembled version or kit for broader adoption among non-engineering labs.

Is the rationale for developing the new method (or application) clearly explained?

Yes

Is the description of the method technically sound?

Yes

Are the conclusions about the method and its performance adequately supported by the findings presented in the article?

Yes

If any results are presented, are all the source data underlying the results available to ensure full reproducibility?

Yes

Are sufficient details provided to allow replication of the method development and its use by others?

Yes

Reviewer Expertise:

My research group in interested in using principles of circadian biology to improve health and wellness.  We utilize mouse models of neurodevelopmental and neurodegenerative diorders to explore these principles.

I confirm that I have read this submission and believe that I have an appropriate level of expertise to confirm that it is of an acceptable scientific standard.

# Response

**Published:** 2025-07-12

**Authors:** Marissa Mueller

**Affiliations:** Physiology Anatomy and Genetics, University of Oxford Department of Physiology Anatomy and Genetics, Oxford, England, UK

We would like to thank the reviewers for their thoughtful, constructive, and insightful feedback on our manuscript. We appreciate the time and effort invested in providing these suggestions, which have been extremely useful in improving this present work. Below, please find point-by-point responses to each comment, wherein edits have been made in the manuscript accordingly with tracked changes. We believe these revisions have strengthened the manuscript and address all concerns raised.  

*The current implementation is only compatible with single housing, limiting its application in studies requiring socially housed animals or high-throughput cohorts. Would it be possible to incorporate RFID or vision-based individual tracking to support social housing.*

**Response **: Most circadian, sleep and metabolic studies require single housing of mice to prevent experimental confounds (e.g. animal interactions and social hierarchies affecting home cage activity, sleep, food access or thermoregulation). As such, the existing *SnackerTracker* system would be compatible with the majority of studies in these fields. However, as the reviewer highlights, studying feeding in group housed animals may be beneficial in other experimental contexts. This would necessitate combining the existing *SnackerTracker* system with video recording (and, for example, AnyMaze tracking) or RFID technology. Whilst this is beyond the scope of the current manuscript, the *SnackerTracker* is fully open-source such that such measures can be incorporated in the future. We have noted this in paragraph 3 of the discussion. Alternatively, the *SnackerTracker* could easily be placed within existing home cage monitoring systems designed for studying social behaviour (e.g. ActualHCA).  

*Crumbling and pellet manipulation not associated with consumption can inflate intake measurements, which may limit precision in studies with subtle feeding changes.  Maybe a special hardened food pellet would improve measurements?*

**Response**: Certainly—this could help circumvent errors due to crumbling. We have noted this in the discussion (later in paragraph 3), citing alternative AIN93M pellets (Research Diet, New Brunswick, NJ, USA) whose hardness can be quantified using a portable device employing the Leeb hardness-testing method (e.g., HMM digital hardness tester, Kern, Stuttgart, BW, DE; Farnell, 3812478). While this could then establish a ‘hardness threshold’ which pellets must exceed for use in *SnackerTrackers*, this could simultaneously introduce systematic error as increasingly hard pellets have been shown to discourage rodent food intake (Ford, 1977). A balance between pellets of sufficient hardness to prevent crumbling, but not so hard as to reduce food intake, should therefore be established. Furthermore, researchers should carefully consider the impact of pellet hardness, and the use of pellets in general, if using animals with known feeding difficulties (e.g., dental occlusion or impaired range of movement). These factors would be worth investigating in future experiments.

*The light-dependent resistor records only relative changes, not absolute irradiance or wavelength, limiting precise circadian photometry applications. Is it possible to improve standardization of light monitoring, possibly by integrating calibrated lux sensors or spectral sensors.*

**Response**: This is an excellent observation, and a topic that our lab has been investigating. While it may be difficult to incorporate increasingly sophisticated calibrated light sensors into the existing *SnackerTracker *configuration, future experiments could easily place cages with *SnackerTrackers *into light-tight chambers (LTCs) equipped with Ocean Optics USB2000 + Spectrophotometers (Ocean Insight, Oxford, United Kingdom), which quantify spectral distributions (Steel et al., 2024). This parallel measurement could greatly improve *SnackerTracker *application in experiments requiring precise light measurements (e.g., for circadian photometry applications). We have mentioned this in paragraph 2 of the discussion.

*Assembly requires technical expertise in soldering, programming, and 3D printing, potentially limiting adoption in labs without engineering support.  Would it be possible to partner with a company to provide a preassembled version or kit for broader adoption among non-engineering labs.*

**Response**: Absolutely, and from our previous experience (Brown et al., 2017), most users wish to purchase a pre-made system rather than building their own. We have therefore partnered with the Oxford Department of Physics’ Electronics design workshop (https://www.physics.ox.ac.uk/about-us/our-facilities-and-services/electronics-design-and-workshop), where we have been quoted as follows (as of July 2025): To order one *SnackerTracker*, a lab would be charged £250.00, and each additional device thereafter would cost £210.00 (including parts and labour). Inquiries may be directed to optec_enquiries@physics.ox.ac.uk. We have added a section to describe this in the discussion (paragraph 16, second-to-last).
